# A Chemical Screening Approach to Identify Novel Key Mediators of Erythroid Enucleation

**DOI:** 10.1371/journal.pone.0142655

**Published:** 2015-11-16

**Authors:** Christina B. Wölwer, Luke B. Pase, Helen B. Pearson, Nathan J. Gödde, Kurt Lackovic, David C. S. Huang, Sarah M. Russell, Patrick O. Humbert

**Affiliations:** 1 Cell Cycle and Cancer Genetics, Peter MacCallum Cancer Centre, Melbourne, Australia; 2 Sir Peter MacCallum Department of Oncology, University of Melbourne, Melbourne, Australia; 3 The Walter and Eliza Hall Institute of Medical Research, Melbourne, Australia; 4 Department of Medical Biology, University of Melbourne, Melbourne, Australia; 5 Immune Signaling Laboratory, Peter MacCallum Cancer Centre, Melbourne, Australia; 6 Centre for Micro-Photonics, Faculty of Engineering and Industrial Sciences, Swinburne University of Technology, Hawthorn, Australia; 7 Department of Pathology, University of Melbourne, Melbourne, Australia; 8 Department of Biochemistry and Molecular Biology, University of Melbourne, Parkville, Victoria, Australia; Southern Illinois University School of Medicine, UNITED STATES

## Abstract

Erythroid enucleation is critical for terminal differentiation of red blood cells, and involves extrusion of the nucleus by orthochromatic erythroblasts to produce reticulocytes. Due to the difficulty of synchronizing erythroblasts, the molecular mechanisms underlying the enucleation process remain poorly understood. To elucidate the cellular program governing enucleation, we utilized a novel chemical screening approach whereby orthochromatic cells primed for enucleation were enriched *ex vivo* and subjected to a functional drug screen using a 324 compound library consisting of structurally diverse, medicinally active and cell permeable drugs. Using this approach, we have confirmed the role of HDACs, proteasomal regulators and MAPK in erythroid enucleation and introduce a new role for Cyclin-dependent kinases, in particular CDK9, in this process. Importantly, we demonstrate that when coupled with imaging analysis, this approach provides a powerful means to identify and characterize rate limiting steps involved in the erythroid enucleation process.

## Introduction

Erythropoiesis involves the gradual progression of hematopoietic stem cells into mature red blood cells. During terminal differentiation, proerythroblasts undergo several differentiation-linked cell divisions producing basophilic, polychromatic and then orthochromatic daughter erythroblasts. The orthochromatic cells ultimately exit from the cell cycle and extrude their nuclei in a process termed erythroid enucleation. The enucleation event involves multiple pathways and shares some similarities with cytokinesis and apoptosis (for reviews see Keerthivasan et al [1] and Ji et al [2]); however, experimental results have been variable due to the difficulty of synchronizing erythroblasts and the inability to exclude indirect effects of inhibitors on the proliferation of earlier erythroblasts. *In vivo* investigations have also been hindered by the necessity of enucleation for organismal survival with genetic knockdown of key genes often associated with either high redundancy or embryonic lethality. Therefore, our understanding of the molecular mechanisms employed during enucleation remains poor.

In this study we isolated orthochromatic erythroblasts poised to enucleate, and exposed them to a compound screen in order to probe the cellular program governing erythroid enucleation. Although chemical inhibitors used in isolation can be misleading due to off target effects, the use of a large and well-validated library can circumvent this issue, and overcome many of the problems of gene targeting by allowing acute blocking of pathways. By applying stringent criteria such as effects from multiple drugs targeting the same pathways, this screening approach can identify new components of previously known pathways, mechanistic insights into cellular process that these pathways affect, and new regulators of enucleation. Here, using this approach we confirm the role of HDACs, proteasomal regulators and MAPK in erythroid enucleation and introduce a new role for Cyclin-dependent kinases, in particular CDK9, in this process.

## Materials and Methods

### Materials

Phenylhydrazine hydrochloride was purchased from Aldrich Chemistry. CD44- PE-Cy7 anti-mouse antibodies and the BrdU Flow Kit were purchased from BD Pharmingen. Ter119-Alexa Fluor 647 anti-mouse antibodies were purchased from Biolegend. Hoechst 33342 was purchased from Invitrogen. Propidium iodide (PI) was purchased from Merck. Dimethyl sulfoxide (DMSO) was purchased from Calbiochem. Rapid Diff stain was purchased from Australian Biostain. Cytochalasin D was purchased from Sigma Aldrich. The 324 compound library and follow up compounds were purchased from Selleck Chemicals.

### Animal experiments and orthochromatic erythroblast isolation

**Fig 1:**
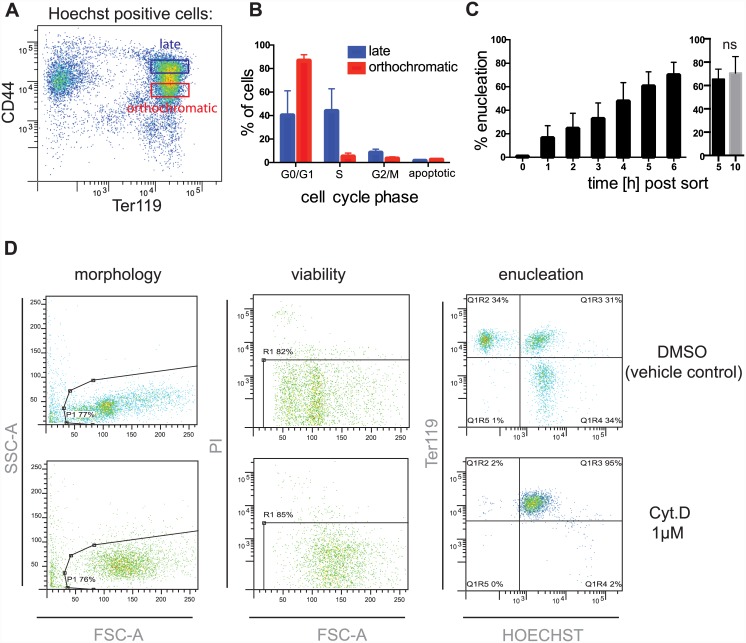
Isolation and characterization of orthochromatic erythroblasts. **(A)** Orthochromatic erythroblasts were isolated (gate highlighted in red) from the spleen by FACS (Aria II) based on their Ter119 versus CD44 expression. Hoechst negative cells were excluded from the sort. **(B)** Cell cycle analysis was performed on the sorted orthochromatic erythroblasts based on *in vitro* BrdU incorporation and 7-AAD staining and compared to the less mature (“late”) erythroblasts (gate highlighted in blue in Fig 1A). **(C)** Enucleation in the sorted population was quantified hourly for 6 hours using. Data are means (+/− SD) of 3 independent experiments. Enucleation rate was also compared between 5 and after 10 hours after the FACS sort. Data are means (+/− SD) of 4 independent experiments. *P< 0.05, **P< 0.01, ***P< 0.001, ****P< 0.0001 (paired student’s t-test). **(D)** The extent of enucleation was assessed by FACS LSR II shown here for negative and positive plate controls (DMSO and Cytochalasin D (Cyt.D) respectively). Propidium iodide (PI) was added to exclude dead cells from the analysis and to reveal potentially cytotoxic effects of the compounds.

All animal procedures were approved by the Peter MacCallum Cancer Centre Animal experimentation Ethics Committee. To induce stress erythropoiesis C57Bl/6 mice at 6–12 weeks of age were administered intraperitoneal injections of phenylhydrazine hydrochloride (60μg/g) on day 0 and day 1 of the experiments. On day 4 of the experiments, cells were harvested from mouse spleens. Using the plunger of a syringe, spleens were pressed through a cell strainer (70μm) and further dissociated into single cell suspensions in PBS (2% FBS) using a 1ml pipette. Cells were stained for Hoechst 33342 (Invitrogen) in pre-warmed alpha-MEM media (containing 10% FBS, 1% Sodium Pyruvate, 1% Glutamax) for 20–30min in a waterbath at 37°C. Cells were subsequently washed and stained for CD44 and Ter119 for 15min on ice. All Hoechst negative (enucleated) cells were excluded from the sort. Orthochromatic erythroblasts were isolated based on their Ter119 (high) and CD44 (low) expression (Fig 1A) by FACS Aria II special order system (BD) using the FACS Diva software (BD). PI was used to exclude dead cells from the sort. Cell cycle analysis was performed by bromodeoxyuridine (BrdU) incorporation *in vitro* according to manufacturers specification. Briefly, BrdU (10μM final concentration) was added to the culture medium (alpha-MEM media supplemented with 10% FBS, 1% Sodium Pyruvate and 1% Glutamax) for a 1h incubation period at 37°C. Cells were then fixed and permeabilized and subsequently treated with DNase. Exposed BrdU was visualized with a FITC-conjugated anti-BrdU antibody. 7-amino-actinomycin D (7-AAD) solution was used for total DNA staining. Cells were analyzed with the FACS LSR II (BD) using the FACS Diva software (BD).

### Compound screen

Orthochromatic erythroblasts (30,000 cells/well) were incubated in 96-well plates (one spleen of a PHZ treated mouse was required per 96-well plate) in the presence of the compounds (1μM) in alpha-MEM media (supplemented with 10%FBS, 1% Sodium Pyruvate and 1% Glutamax) in a final volume of 200μl/well for 5h at 37°C. Each assay plate held negative control wells containing DMSO alone, the compound solvent, as well as positive control wells with cytochalasin D, an inhibitor of erythroid enucleation [3, 4], so as to enable quality control and standardization between each plate. Enucleation was quantified by FACS LSR II (BD) using the FACS Diva software (BD). Net percentage of enucleation was derived by dividing the number of enucleated cells (Ter119^+^/Hoechst^−^) by the sum of enucleated cells and erythroblasts (Ter119^+^/Hoechst^+^), and by subsequently multiplying the quotient by 100. PI was used to exclude dead cells from the analysis. The screen was run in batches and the experiment repeated on a subsequent plate on a separate day. The raw data was normalized using the plate median and transformed into z-scores.

### Cytospins

Nuclei were separated from enucleated reticulocytes mechanically by trituration (gently pipetting up and down). 30,000–60,000 orthochromatic erythroblasts were spun onto slides at 320rpm for 4min. Slides were air-dried before fixing with MeOH, stained with Rapid Diff and quantitated manually under the microscope (Olympus BX-51, 100x/1.40 NA oil objective).

## Results and Discussion

The process of erythroblast enucleation occurs within a 10 minute period [5] and is not synchronized, making it challenging to interpret effects of drugs and shRNA studies. In this study we avoided indirect effects of the compounds on earlier proliferating erythroblasts by isolating orthochromatic erythroblasts, poised to enucleate, to expose to a 324-compound screen.

### Isolation of orthochromatic erythroblasts and enucleation assay

Orthochromatic erythroblasts were isolated from the spleen based on expression of Ter119 and CD44 [6] (Fig 1A) (a cytospin of unsorted spleen cells for comparison is shown in S1 Fig). Cell cycle analysis based on BrdU incorporation confirmed the enrichment of orthochromatic erythroblasts (88.7 ± 3.5% of the isolated erythroblasts were non-proliferating erythroblasts) (Fig 1B). The enucleation rate of the isolated erythroblasts determined hourly increased linearly with time and peaked to around 70 ± 10.5% after 6 hours in media, with no statistical difference observed in enucleation rate between 5 and 10 hours (Fig 1C). Enucleation was quantified by FACS analysis. An example of this analysis is shown in Fig 1D using the negative and positive plate controls. In the presence of the vehicle control (DMSO) erythroblasts enucleate (Ter119 positive, Hoechst negative) but in the presence of Cytochalasin D (1μM) enucleation is blocked (Fig 1D). This is also visible in the forward scatter (FSC) versus side scatter (SSC) dot plots as enucleated cells (reticulocytes) are smaller than orthochromatic erythroblasts. The shift in FSC and SSC profiles may also indicate that the erythroblasts change shape consistent with the known action of the actin inhibitor on cell morphology. Propidium Iodide (PI) was added to exclude dead cells from the analysis and also to help distinguish whether enucleation was blocked because of a specific mechanism being inhibited or as a result of cytotoxicity.

### Chemical screen assay identifies known and novel targets

**Fig 2:**
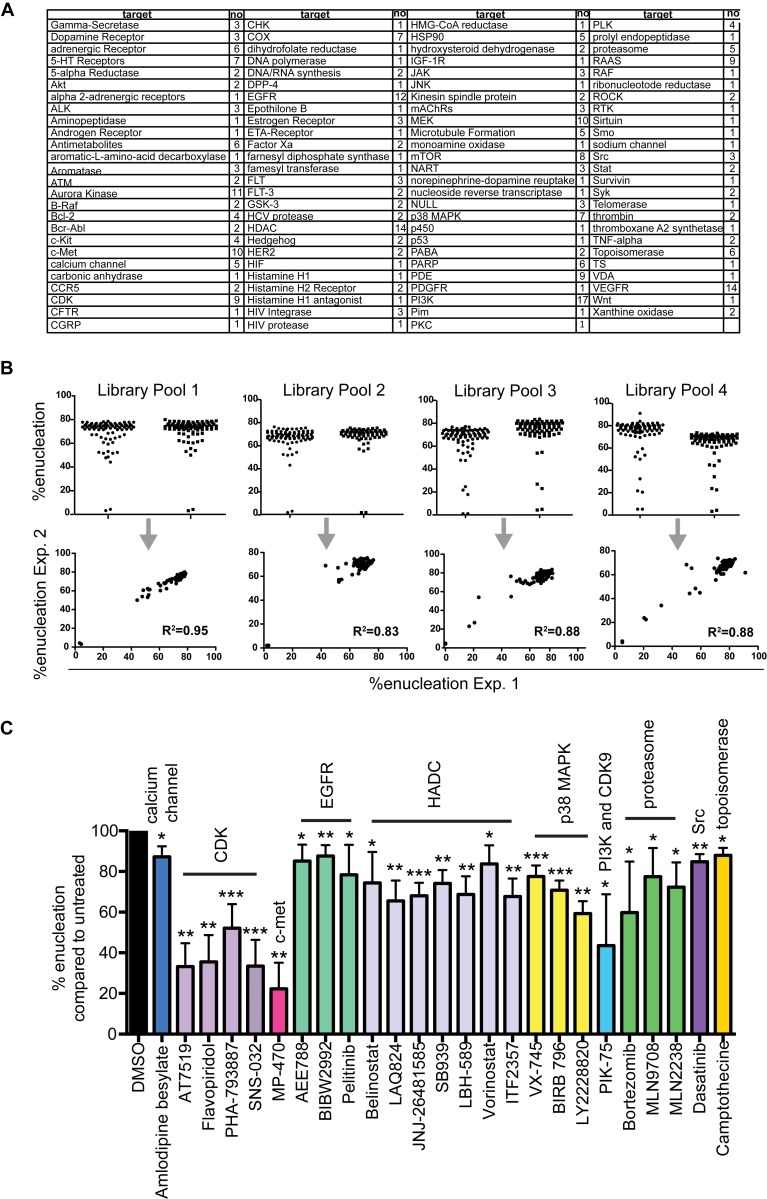
Chemical screen targeting erythroid enucleation. **(A)** List of molecules targeted in the chemical screen. **(B)** For the compound screen orthochromatic erythroblasts were isolated by FACS and subsequently incubated in 96-well plates in the presence of the compounds for 5h. The extent of enucleation was assessed by FACS LSR II. Graphs showing that the raw data between the duplicate assay plates are reproducible. **(C)** Graph showing compounds confirmed to significantly (paired student’s t-test) inhibit enucleation compared to the vehicle control (DMSO). Data are means (+/− SD) of 4 independent experiments. *P< 0.05, **P< 0.01, ***P< 0.001, ****P< 0.0001 (paired student’s t-test).

The Selleck library utilized in our screen contained 324 structurally diverse, medicinally active, cell permeable drugs with multiple drugs targeting the same pathways (Fig 2A). To minimize non-specific effects, each compound was tested at a final concentration of 1μM. The screen was run in batches in 96-well microtiter plates and the experiment repeated on a subsequent plate on a separate day. Correlation coefficients showed that the raw data between the two independent biological repeats was highly reproducible (Fig 2B). The raw data (S1 Table) was normalized and transformed into z-scores (S2 Fig). Compounds demonstrating z-scores <−1 in either replicate were selected as potential hits (S2 Fig) and were tested independently (n = 4) for their ability to inhibit enucleation at 1μM concentrations. Inhibition of enucleation was confirmed for 25 compounds (Fig 2C).

Validating our approach, one of the most significant families of hits centered on HDAC inhibitors (Fig 2C). Previous studies have implicated the importance of chromatin condensation for terminal erythroid differentiation [7–9]; but our findings that HDAC inhibitors affected erythroblasts after exit of the cell cycle, when chromatin condensation should have ceased, suggests that other, non-histone HDAC targets could be involved in the extrusion of the nucleus at this late stage of enucleation. Similarly, we confirm a role for MAPK p38 in erythroid enucleation (Fig 2C). MAPK p38 upregulates p21 and retinoblastoma protein (Rb)[10], both of which are crucial regulators of erythroid enucleation [11, 12]. Our findings suggest that MAPK p38 might play additional post-translational roles in enucleation.

### Proteasome inhibition leads to nuclear segmentation of orthochromatic erythroblasts and decreased enucleation

**Fig 3:**
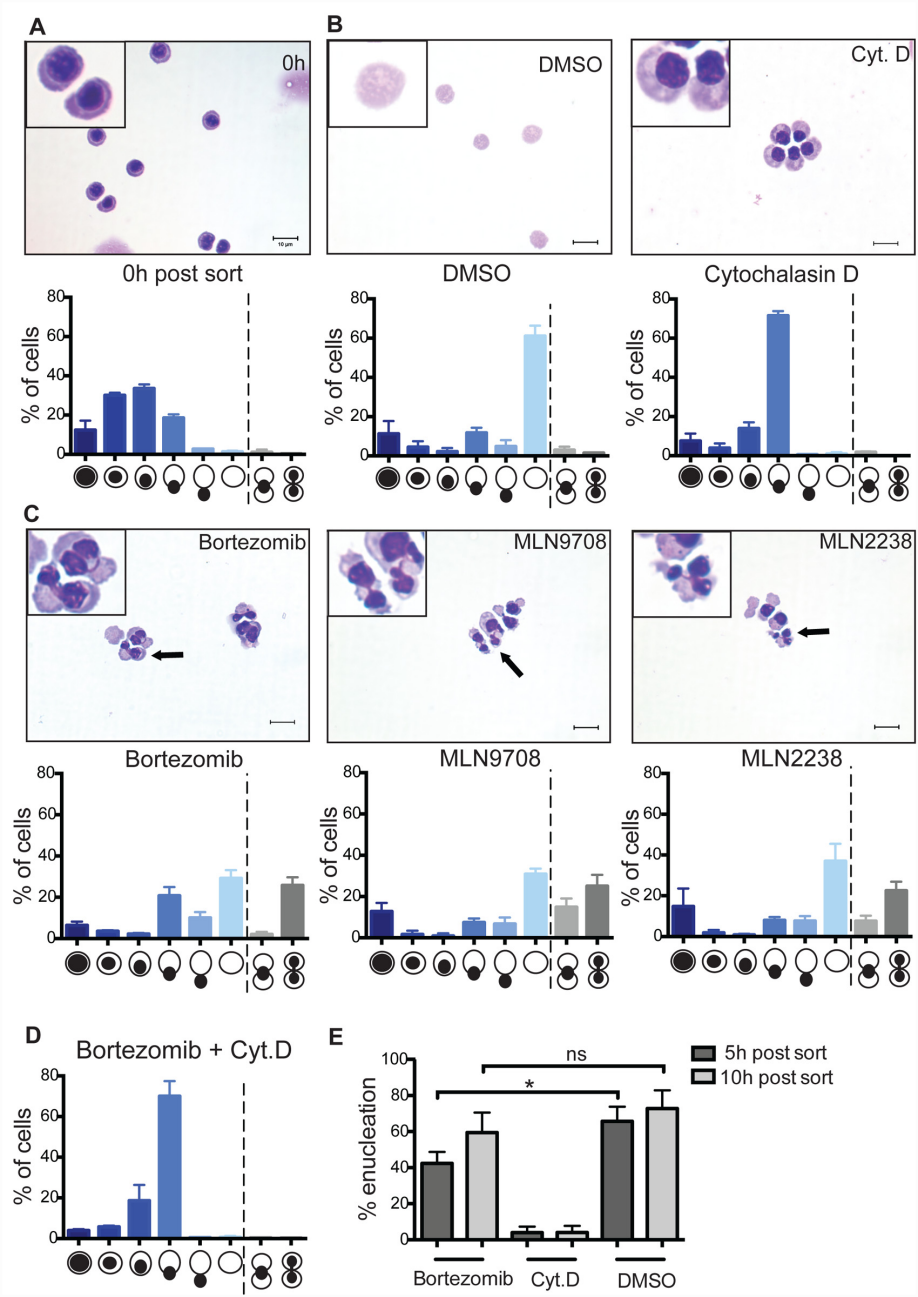
Characterization of a role of the proteasome in erythroid enucleation. **(A)** Orthochromatic erythroblasts were cytospun immediately (0h) after FACS enrichment, Rapid Diff stained and imaged with the Olympus BX-51 microscope (100x/1.40 NA oil objective) using the Spot Advanced software (version 4.7). For quantitative analysis of the cytospins cells were manually examined and assigned a morphological class as per illustration. Data are means (+/− SD) of 4 independent experiments (112–541 cells per experiment were enumerated). **(B)** Orthochromatic erythroblasts were incubated in media containing DMSO (vehicle control) or Cytochalasin D (1μM) for 5h and subsequently cytospun. For quantitative analysis 355–740 cells per experiment were enumerated for the 5h time point in DMSO; 640–712 cells per experiment for the 5h time point in 1μM Cytochalasin D. Data are means (+/− SD) of 4 independent experiments. **(C)** Cytospins and quantitative analysis of orthochromatic erythroblasts treated with the indicated proteasome inhibitors (1μM) for 5h. Data are means (+/− SD) of 3 independent experiments (347–585 cells per experiment treated with Bortezomib; 422–428 cells per experiment treated with MLN9708; 351–698 cells per experiment treated with MLN2238). **(D)** Quantitative analysis of cytospun orthochromatic erythroblasts treated with Bortezomib and Cytochalasin D (1μM each). Data are means (+/− SD) of 3 independent experiments (448–516 cells per experiment treated with Bortezomib in combination with Cyt.D). **(E)** Graph showing percentages of enucleation after 5h and 10h in the presence of Bortezomib (1μM). Data are means (+/− SD) of 3 independent experiments analyzed using FACS LSR II. *P< 0.05, **P< 0.01, ***P< 0.001, ****P< 0.0001 (paired student’s t-test).

Three out of five compounds targeting the proteasome inhibited erythroid enucleation (Fig 2C). A potential requirement for the proteasome in erythroid enucleation has been highlighted in a study by Chen et al [13]. This study showed that enucleation of erythroblasts infected with the anemia-inducing strain of Friend-virus was decreased by up to 40% in the presence of proteasome inhibitors. Our data supports this observation and extends it to determine the cellular effects of proteasome inhibitors on enucleating erythroblasts. Here we show that up to 30% of cytospun enucleating erythroblasts treated with proteasome inhibitors displayed a nuclear segmentation phenotype with the nuclear compartments connected by DNA bridges (Fig 3C). Interestingly, incubating orthochromatic erythroblasts with both proteasome inhibitors and the actin inhibitor Cytochalasin D resulted in arrest of enucleation but prevented segmentation of the nucleus (Fig 3D) suggesting that a proteasome-mediated mechanism may regulate the spatial and/or temporal control of the contractile apparatus required for efficient nuclear extrusion. Alternatively, the Cytochalasin D block may occur “upstream” of the proteasome inhibitor block thus leading to a dominant epistatic phenotype. Furthermore, quantitative analysis revealed that by 10 hours, Bortezomib treated erythroblasts reached normal (vehicle control) levels of enucleation (Fig 3E), indicating that proteasome inhibitors cause a delay rather than an arrest of enucleation.

### Chemical screen identifies CDK9 as a novel regulator of enucleation

**Fig 4:**
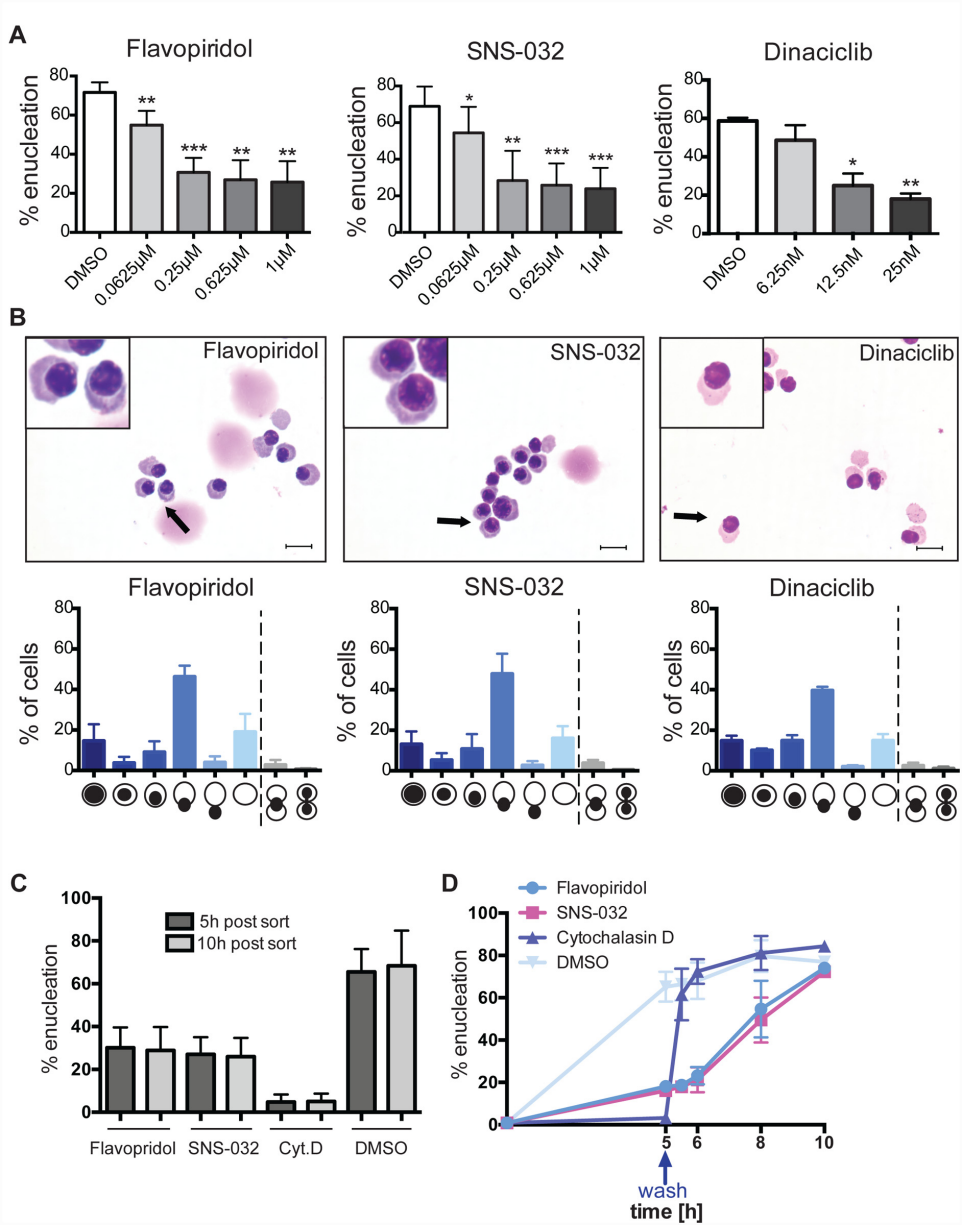
Characterization of a role of CDK activity in erythroid enucleation. Orthochromatic erythroblasts were isolated from the spleen using FACS, and incubated in the presence of the indicated compounds for 5h. **(A)** Graphs showing percentages of enucleation in the presence of the indicated compounds at the indicated concentrations. Data are means (+/− SD) of 3–4 independent experiments analyzed using FACS LSR II (*P< 0.05, **P< 0.01, ***P< 0.001, ****P< 0.0001 (paired student’s t-test)). **(B)** Cytospins and quantitative analysis of orthochromatic erythroblasts treated with the indicated inhibitors for 5h. Data are means (+/− SD) of 3 independent experiments (364–904 cells per experiment treated with Flavopiridol (1μM); 499–730 cells per experiment treated with SNS-032 (1μM); 510–634 cells treated with Dinaciclib (25nM)). Scale bar = 10μm **(C)** Graph showing the percentages of enucleation after 5h and 10h in the presence of the indicated compounds (1μM). Data are means (+/− SD) of 3 independent experiments analyzed using FACS LSR II. **(D)** Compounds were washed out and replaced by media containing the vehicle control DMSO. Enucleation was assessed by FACS LSR II 0.5h, 1h, 3h and 5h after the washout of the indicated compounds (1μM). Data are means (+/− SD) of 3 independent experiments analyzed using FACS LSR II.

What of new targets? Our screen and follow up experiments indicate that CDK activity is important for erythroid enucleation (Fig 2C). Flavopiridol and SNS-032 were among the compounds that inhibited enucleation most efficiently and in a concentration dependent manner (Fig 4A). Since all the CDK inhibitors that resulted in an arrest of enucleation (S3 Fig) are strong inhibitors of both CDK7 and CDK9, Dinaciclib (inhibits CDK9 but not CDK7) was tested for its ability to inhibit enucleation to further specify which of the CDKs is likely to play a role in the extrusion of the nucleus. Dinaciclib inhibited enucleation in a concentration dependent manner at nanomolar concentrations (Fig 4A) confirming that CDK9 activity is the major CDK activity required for nuclear extrusion. Morphological analysis revealed that Flavopiridol, SNS-032 and Dinaciclib arrested extrusion of the nucleus in enucleating cells (Fig 4B). Further supporting the notion that CDK9 activity is important for erythroid enucleation, PIK-75, a dual inhibitor of PI3K and CDK9 [14, 15], but no other PI3K inhibitor, inhibited enucleation and phenocopied the enucleation arrest caused by Flavopiridol and SNS-032 (S4 Fig). Furthermore, percentages of enucleated cells treated with CDK inhibitors did not increase from 5 to 10 hours in culture (Fig 4C), indicating that CDK inhibitors cause an arrest of enucleation rather than a delay. The viability of erythroblasts treated with the CDK inhibitors was not affected with enucleation rates recovering after washout of the CDK inhibitors (Fig 4D).

Interestingly, knockdown of CDK9, the kinase subunit of the transcription factor pTEF-b, results in disruption of primitive erythropoiesis in zebrafish and treatment of human CD34^+^ cells with Flavopiridol, a CDK9 inhibitor, blocked early erythroid differentiation [16]. Our study indicates that CDK9 is also likely to function late in terminal differentiation during nuclear extrusion. CDKs are known to regulate the cell cycle, with CDK9 shown to also play important roles in the regulation of RNA transcription [17]. Because orthochromatic erythroblasts are said to have exited the cell cycle and since chromatin is highly condensed at this stage of development, RNA transcription is curbed [18]. CDKs and in particular CDK9 are likely then to regulate enucleation through other mechanisms. Consistent with this notion, treatment with RNA polymerase II inhibitor α–amanitin did not affect enucleation (S5 Fig). The reversibility of inhibition by CDK inhibitors and other compounds used in this study such as Cytochalasin D provide powerful tools to synchronize erythroblasts at key steps of enucleation and following release of inhibition allow the detailed biochemical and cellular study of the individual steps of enucleation. Combined with proteomics and RNAi studies, this will provide a useful platform to assess the potential mechanism of CDK functions in erythroid enucleation.

### Microtubule and PI3K activity is not required for the actual extrusion of the nucleus during erythroid enucleation

None of the 5 inhibitors targeting microtubules (MTs) inhibited enucleation in our screen. The question of whether MTs are critical for erythroid enucleation has been widely discussed and remains controversial [3, 4, 19, 20]. Similarly, despite evidence that PI3K activity is involved in the establishment of cell polarity in late stage erythroblasts and that PI3K inhibitors delay enucleation [21], we found only one of the 17 compounds targeting PI3Ks to inhibit enucleation (Fig 2C), and this compound, PIK-75, also inhibits CDK9. However, earlier processes that are thought to involve MT and PI3K, such as the movement of the nucleus to one side of the cell, may not have been addressed in our screen since over 33% of the isolated erythroblasts displayed polarized nuclei at 0h already (Fig 3A).

## Conclusion

Taken together, our chemical screening approach allowed us to directly question pathways involved in the extrusion of the nucleus during erythroid enucleation. Of note, the vast majority of enucleation inhibitors (with the notable exception of proteasome inhibitors) show a similar enucleation arrest phenotype at the nuclear extrusion step. This suggests that the protein activities they block either all block the same necessary pathway required to advance past the extrusion step, or alternatively that a common checkpoint is elicited during enucleation when the activity of any of these proteins is aberrant. Finally, our studies implicate CDK9 as an important regulator of enucleation and future work will involve elucidating the critical CDK9 targets required for enucleation. In conclusion, our approach is highly suited to rapidly and directly testing novel classes of inhibitors for effects on enucleation and provides the opportunity of carrying out unbiased chemical screens to identify novel rate limiting steps and targets in the enucleation process.

## Supporting Information

S1 Fig Cytospin of unsorted spleen cells. Image of unsorted, cytospun spleen cells harvested from PHZ treated mice. Scale bar = 20μm.
(PDF)

S2 Fig Analysis of the 324 compound screen targeting erythroid enucleation. Orthochromatic erythroblasts were isolated by FACS and subsequently incubated in 96-well plates in the presence of the compounds for 5h. The extend of enucleation was assessed by FACS. **(A)** Raw data was normalized using the plate median and transformed into z-scores. **(B)** 37 compounds demonstrating z-scores smaller than -1 in either replicate were selected as potential hits and further validated.
(PDF)

S3 Fig CDK inhibitors targeting erythroid enucleation. Graph showing CDK inhibitors (1μM) that resulted in inhibition of enucleation (marked in pink) compared to inhibitors that did not result in inhibition of enucleation (marked in blue).
(PDF)

S4 Fig Characterization of the effects of PI3K/CDK9 inhibitor PIK-75. Orthochromatic erythroblasts were isolated from the spleen using FACS, and incubated in the presence of PIK-75 for 5h. **(A)** Graph showing percentages of enucleation in the presence of PIK-75 at the indicated concentrations. Data are means (+/− SD) of 4 independent experiments analyzed using FACS LSR II (*P< 0.05, **P< 0.01, ***P< 0.001, ****P< 0.0001 (paired student’s t-test)). **(B)** Cytospins and quantitative analysis of orthochromatic erythroblasts treated with PIK-75 for 5h (662–689 cells per experiment treated). Scale bar = 10μm.
(PDF)

S5 Fig Characterization of the effects of RNA-Polymerase II inhibitor. Orthochromatic erythroblasts were isolated from the spleen using FACS, and incubated in the presence of the vehicle control (DMSO) or alpha-amanitin at the indicated concentrations for 5h. Graphs showing percentages of enucleation in. Data are means (+/− SD) of 3–4 independent experiments analyzed using FACS LSR II (*P< 0.05, **P< 0.01, ***P< 0.001, ****P< 0.0001 (paired student’s t-test)).
(PDF)

S1 Table Raw data chemical screen targeting erythroid enucleation. The table lists plate ID, well number, molecular targets, the individual compounds inhibiting these and the corresponding enucleation efficiencies.
(PDF)

## References

[1] Keerthivasan G, Wickrema A, Crispino JD. Erythroblast enucleation. Stem cells international. 2011;2011:139851 Epub 2011/10/19. 10.4061/2011/139851 22007239 PMC3189604

[2] Ji P, Murata-Hori M, Lodish HF. Formation of mammalian erythrocytes: chromatin condensation and enucleation. Trends in cell biology. 2011;21(7):409–15. Epub 2011/05/20. 10.1016/j.tcb.2011.04.003 21592797 PMC3134284

[3] Koury ST, Koury MJ, Bondurant MC. Cytoskeletal distribution and function during the maturation and enucleation of mammalian erythroblasts. The Journal of cell biology. 1989;109(6 Pt 1):3005–13. Epub 1989/12/01. 2574178 10.1083/jcb.109.6.3005 PMC2115945

[4] Keerthivasan G, Small S, Liu H, Wickrema A, Crispino JD. Vesicle trafficking plays a novel role in erythroblast enucleation. Blood. 2010;116(17):3331–40. Epub 2010/07/21. 10.1182/blood-2010-03-277426 20644112 PMC2995360

[5] Yoshida H, Kawane K, Koike M, Mori Y, Uchiyama Y, Nagata S. Phosphatidylserine-dependent engulfment by macrophages of nuclei from erythroid precursor cells. Nature. 2005;437(7059):754–8. Epub 2005/09/30. 10.1038/nature03964 . 16193055

[6] Chen K, Liu J, Heck S, Chasis JA, An X, Mohandas N. Resolving the distinct stages in erythroid differentiation based on dynamic changes in membrane protein expression during erythropoiesis. Proceedings of the National Academy of Sciences of the United States of America. 2009;106(41):17413–8. Epub 2009/10/07. 10.1073/pnas.0909296106 19805084 PMC2762680

[7] Popova EY, Krauss SW, Short SA, Lee G, Villalobos J, Etzell J, et al Chromatin condensation in terminally differentiating mouse erythroblasts does not involve special architectural proteins but depends on histone deacetylation. Chromosome research: an international journal on the molecular, supramolecular and evolutionary aspects of chromosome biology. 2009;17(1):47–64. Epub 2009/01/28. 10.1007/s10577-008-9005-y 19172406 PMC2667965

[8] Ji P, Yeh V, Ramirez T, Murata-Hori M, Lodish HF. Histone deacetylase 2 is required for chromatin condensation and subsequent enucleation of cultured mouse fetal erythroblasts. Haematologica. 2010;95(12):2013–21. Epub 2010/09/09. 10.3324/haematol.2010.029827 20823130 PMC2995558

[9] Zhang L, Flygare J, Wong P, Lim B, Lodish HF. miR-191 regulates mouse erythroblast enucleation by down-regulating Riok3 and Mxi1. Genes & development. 2011;25(2):119–24. Epub 2011/01/05. 10.1101/gad.1998711 21196494 PMC3022257

[10] Schultze SM, Mairhofer A, Li D, Cen J, Beug H, Wagner EF, et al p38alpha controls erythroblast enucleation and Rb signaling in stress erythropoiesis. Cell research. 2012;22(3):539–50. Epub 2011/09/29. 10.1038/cr.2011.159 21946500 PMC3292296

[11] Clark AJ, Doyle KM, Humbert PO. Cell-intrinsic requirement for pRb in erythropoiesis. Blood. 2004;104(5):1324–6. Epub 2004/05/25. 10.1182/blood-2004-02-0618 . 15155463

[12] Walkley CR, Sankaran VG, Orkin SH. Rb and hematopoiesis: stem cells to anemia. Cell division. 2008;3:13 Epub 2008/09/09. 10.1186/1747-1028-3-13 18775080 PMC2562376

[13] Chen CY, Pajak L, Tamburlin J, Bofinger D, Koury ST. The effect of proteasome inhibitors on mammalian erythroid terminal differentiation. Experimental hematology. 2002;30(7):634–9. Epub 2002/07/24. . 12135659 10.1016/s0301-472x(02)00826-3

[14] Thomas D, Powell JA, Vergez F, Segal DH, Nguyen NY, Baker A, et al Targeting acute myeloid leukemia by dual inhibition of PI3K signaling and Cdk9-mediated Mcl-1 transcription. Blood. 2013;122(5):738–48. Epub 2013/06/19. 10.1182/blood-2012-08-447441 . 23775716

[15] Lemke J, von Karstedt S, Abd El Hay M, Conti A, Arce F, Montinaro A, et al Selective CDK9 inhibition overcomes TRAIL resistance by concomitant suppression of cFlip and Mcl-1. Cell death and differentiation. 2014;21(3):491–502. Epub 2013/12/24. 10.1038/cdd.2013.179 24362439 PMC3921597

[16] Bai X, Kim J, Yang Z, Jurynec MJ, Akie TE, Lee J, et al TIF1gamma controls erythroid cell fate by regulating transcription elongation. Cell. 2010;142(1):133–43. Epub 2010/07/07. 10.1016/j.cell.2010.05.028 20603019 PMC3072682

[17] Fischer PM, Gianella-Borradori A. Recent progress in the discovery and development of cyclin-dependent kinase inhibitors. Expert opinion on investigational drugs. 2005;14(4):457–77. Epub 2005/05/11. 10.1517/13543784.14.4.457 . 15882121

[18] Xu Y, Leung CG, Lee DC, Kennedy BK, Crispino JD. MTB, the murine homolog of condensin II subunit CAP-G2, represses transcription and promotes erythroid cell differentiation. Leukemia: official journal of the Leukemia Society of America, Leukemia Research Fund, UK. 2006;20(7):1261–9. Epub 2006/05/05. 10.1038/sj.leu.2404252 . 16673016

[19] Chasis JA, Prenant M, Leung A, Mohandas N. Membrane assembly and remodeling during reticulocyte maturation. Blood. 1989;74(3):1112–20. Epub 1989/08/15. . 2752157

[20] Konstantinidis DG, Pushkaran S, Johnson JF, Cancelas JA, Manganaris S, Harris CE, et al Signaling and cytoskeletal requirements in erythroblast enucleation. Blood. 2012;119(25):6118–27. Epub 2012/03/31. 10.1182/blood-2011-09-379263 22461493 PMC3383020

[21] Wang J, Ramirez T, Ji P, Jayapal SR, Lodish HF, Murata-Hori M. Mammalian erythroblast enucleation requires PI3K-dependent cell polarization. Journal of cell science. 2012;125(Pt 2):340–9. Epub 2012/02/15. 10.1242/jcs.088286 22331356 PMC3283871

