# Supplementary figures and images for: A Chemical Screening Approach to Identify Novel Key Mediators of Erythroid Enucleation
Source: PLoS One. 2015 Nov 16;10(11):e0142655. doi: 10.1371/journal.pone.0142655 (PMC4646491; doi:10.1371/journal.pone.0142655)

**S1 Fig**

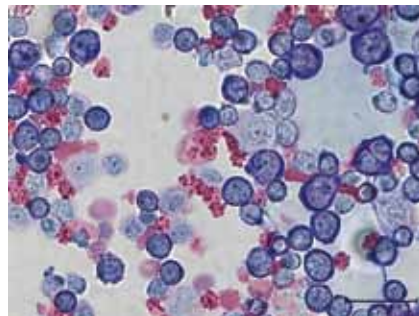

Supplement: S1 Fig — Image of unsorted, cytospun spleen cells harvested from PHZ treated mice. Scale bar = 20μm. (PDF) [file pone.0142655.s001.pdf]

S3 Fig

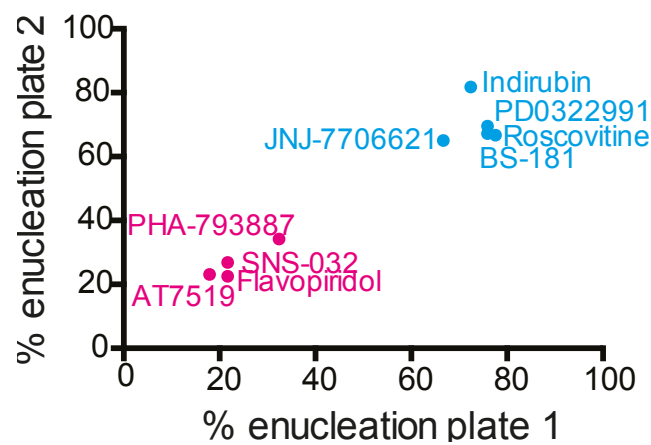

Supplement: S3 Fig — Graph showing CDK inhibitors (1μM) that resulted in inhibition of enucleation (marked in pink) compared to inhibitors that did not result in inhibition of enucleation (marked in blue). (PDF) [file pone.0142655.s003.pdf]

S4 Fig

A

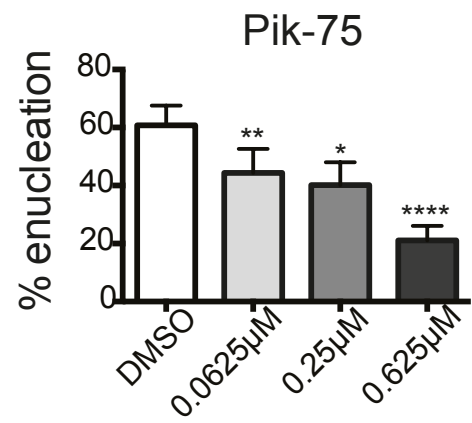

B

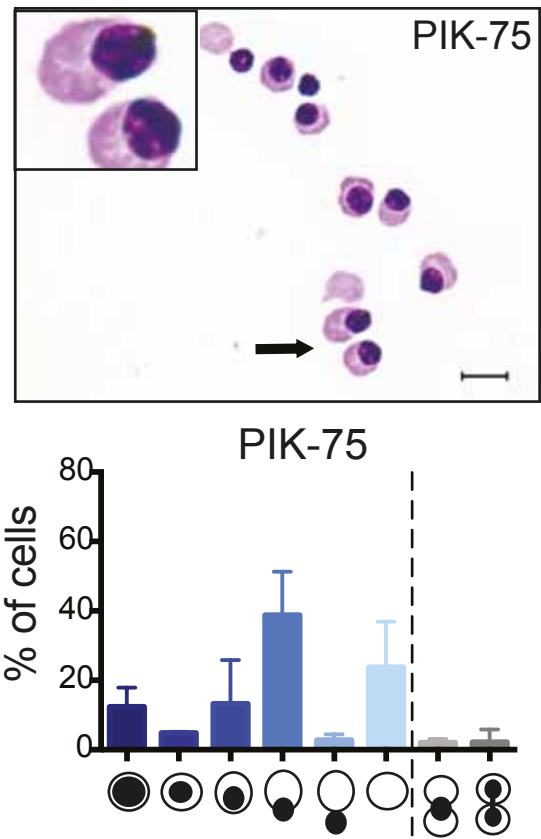

Supplement: S4 Fig — Orthochromatic erythroblasts were isolated from the spleen using FACS, and incubated in the presence of PIK-75 for 5h. (A) Graph showing percentages of enucleation in the presence of PIK-75 at the indicated concentrations. Data are means (+/− SD) of 4 independent experiments analyzed using FACS LSR II (*P< 0.05, **P< 0.01, ***P< 0.001, ****P< 0.0001 (paired student’s t-test)). (B) Cytospins and quantitative analysis of orthochromatic erythroblasts treated with PIK-75 for 5h (662–689 cells per experiment treated). Scale bar = 10μm. (PDF) [file pone.0142655.s004.pdf]

S5 Fig

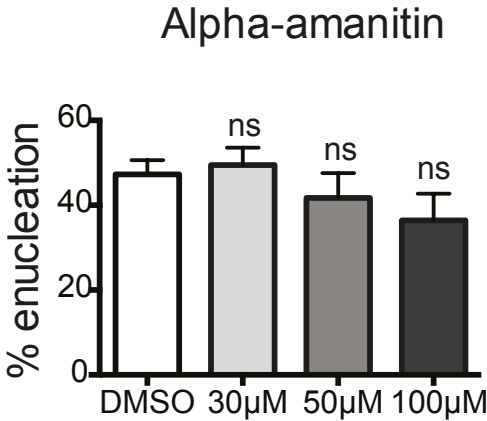

Supplement: S5 Fig — Orthochromatic erythroblasts were isolated from the spleen using FACS, and incubated in the presence of the vehicle control (DMSO) or alpha-amanitin at the indicated concentrations for 5h. Graphs showing percentages of enucleation in. Data are means (+/− SD) of 3–4 independent experiments analyzed using FACS LSR II (*P< 0.05, **P< 0.01, ***P< 0.001, ****P< 0.0001 (paired student’s t-test)). (PDF) [file pone.0142655.s005.pdf]
