# Supplementary material for: A Chemical Screening Approach to Identify Novel Key Mediators of Erythroid Enucleation
Source: PLoS One. 2015 Nov 16;10(11):e0142655. doi: 10.1371/journal.pone.0142655 (PMC4646491; doi:10.1371/journal.pone.0142655)
Supplement: S2 Fig — Orthochromatic erythroblasts were isolated by FACS and subsequently incubated in 96-well plates in the presence of the compounds for 5h. The extend of enucleation was assessed by FACS. (A) Raw data was normalized using the plate median and transformed into z-scores. (B) 37 compounds demonstrating z-scores smaller than -1 in either replicate were selected as potential hits and further validated. (PDF) [file pone.0142655.s002.pdf]

## S2 Fig

A

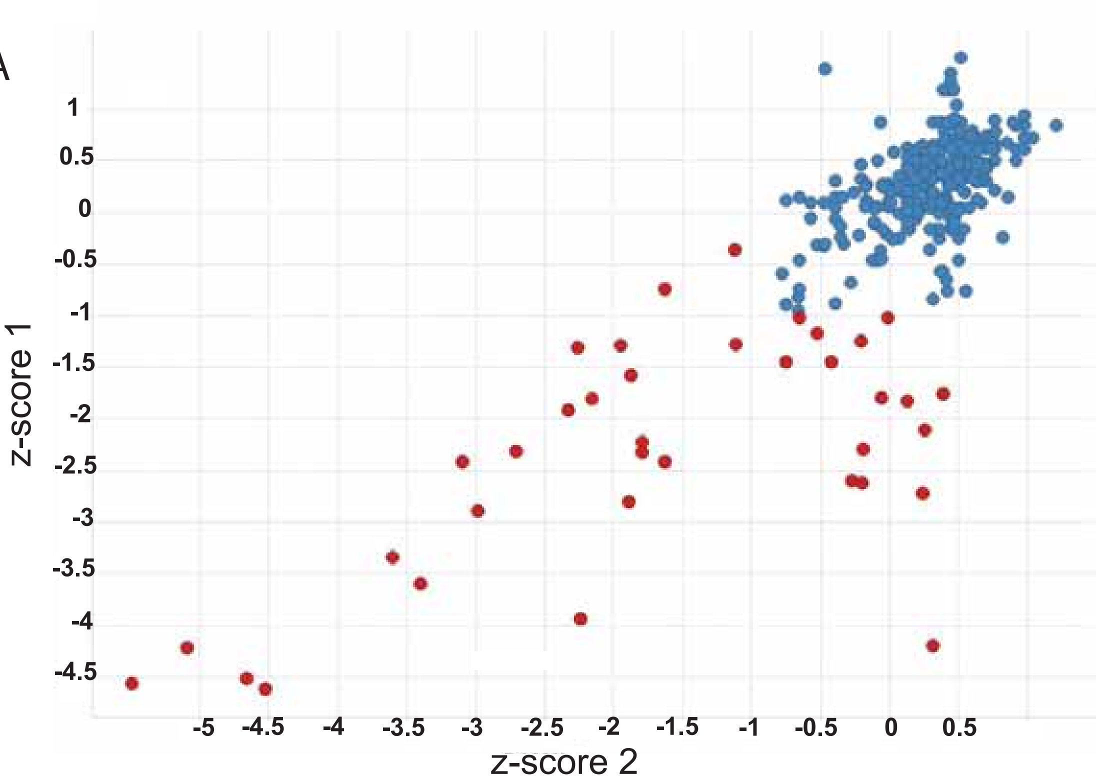


B

| target | compound | z-score 1 | z-score 2 |
|---|---|---|---|
| Bcl-2 | Obatoclax Mesyl. | -1.92 | -2.33 |
| calcium channel | Felodipine | -2.72 | 0.24 |
| calcium channel | Amlodipine besyl. | -2.1 | 0.26 |
| CDK | AT7519 | -4.57 | -5.49 |
| CDK | SNS-032 | -4.21 | -5.09 |
| CDK | Flavopiridol | -4.51 | -4.66 |
| CDK | PHA-793887 | -3.6 | -3.4 |
| c-Met | MP-470 | -3.94 | -2.24 |
| EGFR | AEE788 | -2.29 | -0.19 |
| EGFR | BIBW2992 | -1.02 | -0.02 |
| EGFR | Pelitinib | -0.36 | -1.12 |
| HDAC | Belinostat | -2.31 | -2.71 |
| HDAC | CUDC-101 | -0.74 | -1.63 |
| HDAC | LAQ824 | -3.34 | -3.6 |
| HDAC | JNJ-26481585 | -2.89 | -2.98 |
| HDAC | SB939 | -2.22 | -1.79 |
| HDAC | LBH-589 | -2.42 | -3.1 |
| HDAC | Vorinostat | -2.61 | -0.28 |
| HDAC | ITF2357 | -1.31 | -2.26 |
| p38 MAPK | VX-702 | -1.29 | -1.95 |
| p38 MAPK | VX-745 | -1.45 | -0.75 |
| p38 MAPK | BIRB 796 | -1.02 | -0.65 |
| p38 MAPK | LY2228820 | -1.57 | -1.87 |
| PARP | AG-014699 | -1.8 | -0.05 |
| PI3K | PIK-75 | -4.61 | -4.53 |
| Pim | SGI-1776 | -4.19 | 0.31 |
| PLK | BI 2536 | -1.45 | -0.42 |
| proteasome | Bortezomib | -2.8 | -1.89 |
| proteasome | MLN9708 | -2.32 | -1.79 |
| proteasome | MLN2238 | -2.42 | -1.63 |
| RAAS | Ramipril | -1.75 | 0.39 |
| Src | Bosutinib | -1.25 | -0.21 |
| Src | Dasatinib | -1.17 | -0.53 |
| topoisomerase | Irinotecan | -1.28 | -1.11 |
| topoisomerase | Mitoxantrone | -2.62 | -0.2 |
| topoisomerase | Camptothecine | -1.83 | 0.13 |
| VEGFR | AP24534 | -1.81 | -2.16 |
