# Supplementary material for: A Chemical Screening Approach to Identify Novel Key Mediators of Erythroid Enucleation
Source: PLoS One. 2015 Nov 16;10(11):e0142655. doi: 10.1371/journal.pone.0142655 (PMC4646491; doi:10.1371/journal.pone.0142655)
Supplement: S1 Table — The table lists plate ID, well number, molecular targets, the individual compounds inhibiting these and the corresponding enucleation efficiencies. (PDF) [file pone.0142655.s006.pdf]

S1 Table. Raw Data Chemical Screen

Plate ID	Well	compound	target	%enucleation
Plate 00016365	A1	Temsirolimus	mTOR	71.42857
Plate 00016365	A10	Maraviroc(Selzentry)	CCR5(cellular coreceptor 5)	75
Plate 00016365	A11	Danoprevir(ITMN-191)	Proteasome	75.92593
Plate 00016365	A12	Lenalidomide	TNF-alpha	76.36364
Plate 00016365	A2	TGX-221	PI3K	71.92982
Plate 00016365	A3	Etoposide(Etopophos)	Topoisomerase	73.58491
Plate 00016365	A4	VX-702	p38 MAPK	60.65574
Plate 00016365	A5	LY294002	PI3K	68.42105
Plate 00016365	A6	Cytarabine(Cytosar-U)	DNA polymerase	73.21429
Plate 00016365	A7	Belinostat(PXD101)	HDAC	52.38095
Plate 00016365	A8	S31-201	STAT3	71.42857
Plate 00016365	A9	CUDC-101	HDAC	65
Plate 00016365	B1	Decitabine	Antimetabolites	74.54546
Plate 00016365	B10	Ispinesib mesilate	Kinesin spindle protein	71.42857
Plate 00016365	B11	Ritonavir	HIV protease	74.54546
Plate 00016365	B12	MS-275	HDAC	76.36364
Plate 00016365	B2	IC-87114	PI3K	73.68421
Plate 00016365	B3	SB 431542	ALK	63.7931
Plate 00016365	B4	PLX-4720	B-Raf	75
Plate 00016365	B5	SB 216763	GSK-3	73.68421
Plate 00016365	B6	EX 527	Sirtuin	73.68421
Plate 00016365	B7	BIIB021	HSP90	67.24138
Plate 00016365	B8	LAQ824	HDAC	44.11765
Plate 00016365	B9	Raltegravir	HIV Integrase	75.4386
Plate 00016365	C1	AS-605240	PI3K	75.4386
Plate 00016365	C10	MLN9708	Proteasome	52.30769
Plate 00016365	C11	BMS-790052	HCV protease	65.51724
Plate 00016365	C12	NVP-AUY922	HSP90	67.24138
Plate 00016365	C2	ZSTK474	PI3K	72.72727
Plate 00016365	C3	PD98059	MEK	71.92982
Plate 00016365	C4	Irinotecan	topoisomerase	60.71429
Plate 00016365	C5	BIX 02189	MEK	71.92982
Plate 00016365	C6	Pomalidomide	TNF-alpha	74.54546
Plate 00016365	C7	AG14361	PARP	73.68421
Plate 00016365	C8	JNJ-26481585	HDAC	47.76119
Plate 00016365	C9	VX-222	HCV protease	78.18182
Plate 00016365	D1	Cladribine	Antimetabolites	77.77778
Plate 00016365	D10	BMS-708163	¦Ã-secretase	74.54546
Plate 00016365	D11	ABT-888	PARP	73.68421
Plate 00016365	D12	Trichostatin A	HDAC	63.33333
Plate 00016365	D2	GSK429286A	ROCK	68.96552
Plate 00016365	D3	Fludarabine(Fludara)	Antimetabolites	74.54546
Plate 00016365	D4	Paclitaxel(Taxol)	Microtubule Formation	67.24138
Plate 00016365	D5	GDC-0941	PI3K	71.92982
Plate 00016365	D6	LY500307	Er¦Â	75
Plate 00016365	D7	JNJ 26854165	p53	73.21429
Plate 00016365	D8	MGCD0103(Mocetinostat)	HDAC	75
Plate 00016365	D9	Elvitegravir	HIV Integrase	76.78571
Plate 00016365	E1	Clofarabine	ribonucleotide reductase	75.47169
Plate 00016365	E10	Dutasteride(Avodart)	5¦Á-reductase	76.78571
Plate 00016365	E11	AZD2281(Olaparib)	PARP	75.47169
Plate 00016365	E12	Flucytosine(Ancobon)	DNA/RNA synthesis	76.78571

Plate 00016365	E2	VX-745	p38 MAPK	59.32203
Plate 00016365	E3	2-Methoxyestradiol	HIF	73.68421
Plate 00016365	E4	CI-1040 (PD184352)	MEK	76.36364
Plate 00016365	E5	SB 202190	p38 MAPK	64.40678
Plate 00016365	E6	BSI-201	PARP	77.77778
Plate 00016365	E7	SB939	HDAC	53.125
Plate 00016365	E8	BMS-707035	HIV-1 Integrase(IN)	75.92593
Plate 00016365	E9	RO4929097	Y-Secretase	76.78571
Plate 00016365	F1	TG100-115	PI3K	76.36364
Plate 00016365	F10	17-AAG(Geldanamycin)	HSP90	71.42857
Plate 00016365	F11	Bortezomib	proteasome	48.48485
Plate 00016365	F12	Droxinostat	HDAC	77.77778
Plate 00016365	F2	XL147	PI3K	75
Plate 00016365	F3	PIK-93	PI3K	75
Plate 00016365	F4	PD0325901	MEK	77.77778
Plate 00016365	F5	SB 203580	p38 MAPK	75
Plate 00016365	F6	Cryptotanshinone	Stat	76.78571
Plate 00016365	F7	MLN2238	Proteasome	51.5625
Plate 00016365	F8	Cyclopamine	Hedgehog	78.57143
Plate 00016365	F9	NVP-BEP800	HSP90	71.92982
Plate 00016365	G1	PIK-90	PI3K	72.72727
Plate 00016365	G10	Atazanavir(BMS-232632)	Proteasome	76.78571
Plate 00016365	G11	LBH-589(Panobinostat)	HDAC	51.5625
Plate 00016365	G12	Ketoprofen(Actron)	COX	75.92593
Plate 00016365	G2	KU-0063794	mTOR	76.36364
Plate 00016365	G3	KU-55933	ATM	73.21429
Plate 00016365	G4	SP600125	JNK	74.54546
Plate 00016365	G5	Sodium valproate	HDAC	75.92593
Plate 00016365	G6	MC1568	HDAC	75.92593
Plate 00016365	G7	Semagacestat	Y-Secretase	76.78571
Plate 00016365	G8	GDC-0449(Vismodegib)	Hedgehog	75
Plate 00016365	G9	Tipifarnib	farnesyltransferase(Ftase)	77.77778
Plate 00016365	H1	DMSO	vehicle control	76.36364
Plate 00016365	H2	DMSO	vehicle control	75.92593
Plate 00016365	H3	Cytochalasin D	actin	4.347826
Plate 00016365	H4	Cytochalasin D	actin	3.225806
Plate 00016366	A1	Mizoribine	DNA/RNA synthesis	66.10169
Plate 00016366	A10	SGI-1776	Pim	43.10345
Plate 00016366	A11	Mitoxantrone	Topoisomerase	52.3
Plate 00016366	A2	Vorinostat	HDAC	52.38095
Plate 00016366	A3	Piroxicam	COX	68.96552
Plate 00016366	A4	Chlorprothixene	5-HT	68.42105
Plate 00016366	A5	Fluvastatin Sodium	HMG-CoA reductase	70.17544
Plate 00016366	A6	Manidipine	calcium channel	67.24138
Plate 00016366	A7	Eplerenone	RAAS	62.71186
Plate 00016366	A8	Linezolid	NULL	66.66666
Plate 00016366	A9	Sulfanilamide	PABA	68.42105
Plate 00016366	B1	Letrozole	Aromatase	70.17544
Plate 00016366	B10	AEE788	EGFR	54.23729
Plate 00016366	B11	ABT-869	RTK	66.07143
Plate 00016366	B2	Ibuprofen	COX	68.42105
Plate 00016366	B3	ABT-737	Bcl-2	67.85714
Plate 00016366	B4	Febuxostat	XAO	71.42857
Plate 00016366	B5	Rivaroxaban(Xarelto)	Factor Xa	70.90909
Plate 00016366	B6	BIBR-1048	thrombin	70.90909

Plate 00016366	B7	Ramipril	RAAS	57.37705
Plate 00016366	B8	Felodipine	calcium channel	51.72414
Plate 00016366	B9	Carbamazepine(Carbatrol)	sodium channel	69.09091
Plate 00016366	C1	Anastrozole(Arimidex)	Aromatase	66.66666
Plate 00016366	C10	AG-490	JAK	74.54546
Plate 00016366	C11	BEZ235(NVP-BEZ235)	mTOR	66.66666
Plate 00016366	C2	XAV-939	Wnt	65.51724
Plate 00016366	C3	ABT-263(Navitoclax)	Bcl-2	64.28571
Plate 00016366	C4	Trilostane	hydroxysteroid dehydrogenase	67.85714
Plate 00016366	C5	Baicalin	prolyl endopeptidase	68.42105
Plate 00016366	C6	Icariin	PDE	72.72727
Plate 00016366	C7	Irbesartan(Avapro)	RAAS	67.24138
Plate 00016366	C8	Zalcitabine	NART	70.17544
Plate 00016366	C9	Pyrimethamine	dihydrofolate reductase(DHFR)	68.42105
Plate 00016366	D1	Exemestane	aromatase enzyme	63.7931
Plate 00016366	D10	BIBW2992(Tovok)	EGFR	61.66667
Plate 00016366	D11	PI-103	PI3K	68.42105
Plate 00016366	D2	Bicalutamide(Casodex)	androgen receptor	72.72727
Plate 00016366	D3	Allopurinol	xanthine oxidase	66.66666
Plate 00016366	D4	Pimobendan(Vetmedin)	PDE	67.85714
Plate 00016366	D5	Apixaban	Factor Xa	72.22222
Plate 00016366	D6	S-(+)-Rolipram	PDE	74.54546
Plate 00016366	D7	Valsartan	RAAS	69.64286
Plate 00016366	D8	Cilostazol	PDE	73.68421
Plate 00016366	D9	Dipyridamole	PDE	71.42857
Plate 00016366	E1	Finasteride	5¦Á-reductase	72.72727
Plate 00016366	E10	Sulfamethoxazole	PABA	75.47169
Plate 00016366	E2	Fulvestrant	estrogen receptor	70.17544
Plate 00016366	E3	Stavudine	NART	66.66666
Plate 00016366	E4	Nisoldipine	calcium channel	72.72727
Plate 00016366	E5	BIBR 953	thrombin	75
Plate 00016366	E6	Luteolin(Luteolol)	PDE	74.54546
Plate 00016366	E7	Telmisartan	RAAS	66.66666
Plate 00016366	E8	Amisulpride	Dopamine Receptor	76.36364
Plate 00016366	E9	Clozapine	5-HT1C receptor	70.90909
Plate 00016366	F1	CHR-2797	Aminopeptidase N(APN)	66.66666
Plate 00016366	F10	Apigenin	P450	71.42857
Plate 00016366	F2	INO-1001	PARP	74.54546
Plate 00016366	F3	Emtricitabine	nucleoside reverse transcriptase	69.64286
Plate 00016366	F4	Tadalafil(Cialis)	PDE	64.91228
Plate 00016366	F5	LDE225	Smo	63.15789
Plate 00016366	F6	Nizatidine	histamine H2-receptor	70.17544
Plate 00016366	F7	Carbidopa	aromatic-L-amino-acid decarboxylase	72.22222
Plate 00016366	F8	Naftopidil	¦Á1-adrenergic receptor	66.66666
Plate 00016366	F9	GSK1059615	PI3K	67.85714
Plate 00016366	G1	TW-37	Bcl-2	63.15789
Plate 00016366	G10	JNJ-7706621	CDK	66.66666
Plate 00016366	G2	Acetaminophen(Tylenol)	COX	67.85714
Plate 00016366	G3	Tenofovir	NART	67.85714
Plate 00016366	G4	VX-770	CFTR	68.42105
Plate 00016366	G5	Vardenafil(Vivanza)	PDE	72.72727
Plate 00016366	G6	Enalaprilat	RAAS	67.85714
Plate 00016366	G7	Ozagrel	thromboxane A2 synthetase	66.10169
Plate 00016366	G8	Cilnidipine	calcium channe	64.28571
Plate 00016366	G9	Cilomilast(SB-207499)	PDE	69.64286

Plate 00016366	H1	DMSO	vehicle control	60.34483
Plate 00016366	H2	DMSO	vehicle control	66.66667
Plate 00016366	H3	Cytochalasin D	actin	2.150538
Plate 00016366	H4	Cytochalasin D	actin	3.225806
Plate 00016367	A1	BAY 73-4506(Regorafenib)	c-Kit	57.8125
Plate 00016367	A10	Axitinib	VEGFR	68.96552
Plate 00016367	A11	KU-60019	ATM	65.57377
Plate 00016367	A12	AZD6244(Selumetinib)	MEK	70.68965
Plate 00016367	A2	Dasatinib	SRC	54.6875
Plate 00016367	A3	Cediranib(AZD2171)	VEGFR	68.33334
Plate 00016367	A4	Raltitrexed(Tomudex)	Antimetabolites	70.68965
Plate 00016367	A5	TSU-68	VEGFR	73.68421
Plate 00016367	A6	Docetaxel	Microtubule Formation	71.18644
Plate 00016367	A7	Vandetanib	VEGFR	74.13793
Plate 00016367	A8	SU11274(PKI-SU11274)	c-Met	63.93443
Plate 00016367	A9	ABT-751	Microtubule Formation	68.33334
Plate 00016367	B1	MGCD-265	c-Met	63.33333
Plate 00016367	B10	JNJ-38877605	c-Met	74.13793
Plate 00016367	B11	Enzastaurin	PKC	75.86207
Plate 00016367	B12	Imatinib(STI571)	PDGFR	67.24138
Plate 00016367	B2	Camptothecine	Topoisomerase	47.54099
Plate 00016367	B3	ZM-447439	Aurora Kinase	67.79661
Plate 00016367	B4	Epothilone B(EPO906)	Epothilone B	74.13793
Plate 00016367	B5	MP-470	c-Met	24.67533
Plate 00016367	B6	XL184	VEGFR	69.49152
Plate 00016367	B7	BIBR1532	Telomerase	73.68421
Plate 00016367	B8	AZD7762	CHK	70
Plate 00016367	B9	LY2784544	JAK	66.66666
Plate 00016367	C1	Bosutinib(SKI-606)	Src	53.84615
Plate 00016367	C10	AC-220	FLT	71.18644
Plate 00016367	C11	Hesperadin	Aurora kinase	60.9375
Plate 00016367	C12	AT7867	Akt	65.07937
Plate 00016367	C2	Zibotentan(ZD4054)	ETA-receptor	71.92982
Plate 00016367	C3	Nilotinib	Bcr-Abl	68.33334
Plate 00016367	C4	Vinorelbine(Navelbine)	p38 MAPK	69.49152
Plate 00016367	C5	Tandutinib (MLN518)	FLT-3	68.96552
Plate 00016367	C6	Neratinib	HER2	62.29508
Plate 00016367	C7	Fludarabine Phosphate	Antimetabolites	74.57627
Plate 00016367	C8	XL880(GSK1363089)	c-Met	68.96552
Plate 00016367	C9	BIRB 796	p38 MAPK	56.25
Plate 00016367	D1	PHA-739358(Danusertib)	Aurora Kinase	70
Plate 00016367	D10	Quercetin(Sophoretin)	PI3K	70
Plate 00016367	D11	NVP-TAE684	ALK	71.18644
Plate 00016367	D12	AZD6482	PI3K	71.92982
Plate 00016367	D2	WYE-354	mTOR	73.68421
Plate 00016367	D3	AT7519	CDK	17.85714
Plate 00016367	D4	CCT129202	Aurora kinase	75
Plate 00016367	D5	Indirubin	CDK	72.4138
Plate 00016367	D6	SB 525334	ALK	73.68421
Plate 00016367	D7	AZD8055	mTOR	70.68965
Plate 00016367	D8	PF-04217903	c-Met	68.42105
Plate 00016367	D9	Deforolimus(MK-8669)	mTOR	75.4386
Plate 00016367	E1	BIBF1120(Vargatef)	VEGFR	67.74194
Plate 00016367	E10	NPI-2358	VDA	67.24138
Plate 00016367	E11	CP-724714	HER2	70

Plate 00016367	E12	Epothilone A	Microtubule Formation	66.10169
Plate 00016367	E2	XL765	PI3K	72.88136
Plate 00016367	E3	Masitinib(AB1010)	c-Kit	68.96552
Plate 00016367	E4	GSK461364	PLK	70.68965
Plate 00016367	E5	Capecitabine(Xeloda)	Antimetabolites	74.13793
Plate 00016367	E6	AP24534	VEGFR	47.76119
Plate 00016367	E7	RAF265	RAF	69.49152
Plate 00016367	E8	R406(free base)	Syk	75.4386
Plate 00016367	E9	AZD1152-HQPA(Barasertib)	Aurora Kinase	70.68965
Plate 00016367	F1	AT9283	Bcr-Abl	66.66666
Plate 00016367	F10	PIK-293	PI3K	75.4386
Plate 00016367	F11	AZD0530(Saracatinib)	SRC	73.68421
Plate 00016367	F12	PD318088	MEK	70.68965
Plate 00016367	F2	WZ4002	EGFR	70
Plate 00016367	F3	Gefitinib(Iressa)	EGFR	68.96552
Plate 00016367	F4	PF-2341066	c-Met	74.13793
Plate 00016367	F5	CHIR-99021	GSK-3	66.10169
Plate 00016367	F6	CYC116	Aurora Kinase	67.21311
Plate 00016367	F7	Everolimus(RAD001)	mTOR	72.4138
Plate 00016367	F8	VX-680	Aurora Kinase	77.19299
Plate 00016367	F9	Aurora A Inhibitor I	Aurora Kinase	68.96552
Plate 00016367	G1	CI-1033(Canertinib)	EGFR	59.32203
Plate 00016367	G10	Pemetrexed disodium	TS	73.21429
Plate 00016367	G11	SNS-032(BMS-387032)	CDK	21.68675
Plate 00016367	G12	Rapamycin(Sirolimus)	mTOR	74.54546
Plate 00016367	G2	MLN8237	Aurora	73.68421
Plate 00016367	G3	AZD1480	JAK	76.78571
Plate 00016367	G4	BI 2536	PLK	51.66667
Plate 00016367	G5	BMS 777607	c-Met	73.68421
Plate 00016367	G6	AV-951(Tivozanib)	VEGFR	72.4138
Plate 00016367	G7	PHA-680632	Aurora Kinase	76.78571
Plate 00016367	G8	GDC-0879	B-Raf	74.13793
Plate 00016367	G9	Ki8751	VEGFR	71.92982
Plate 00016367	H1	DMSO	vehicle control	76.78571
Plate 00016367	H2	DMSO	vehicle control	72.88136
Plate 00016367	H3	Cytochalasin D	actin	1.052632
Plate 00016367	H4	Cytochalasin D	actin	1.06383
Plate 00016368	A1	ENMD-2076	Flt	69.49152
Plate 00016368	A10	Erlotinib Hydrochloride	EGFR	73.68421
Plate 00016368	A11	OSI-930	c-Kit	74.57627
Plate 00016368	A12	Granisetron Hydrochloride	5-HT receptor	80
Plate 00016368	A2	Sotalol hydrochloride	Beta-1 adrenergic receptor	75
Plate 00016368	A3	Clemastine fumarate	histamine H1 antagonist	75.86207
Plate 00016368	A4	Diclofenac sodium	COX	77.19299
Plate 00016368	A5	Fasudil HCl	ROCK	77.19299
Plate 00016368	A6	WZ3146	EGFR	73.68421
Plate 00016368	A7	Benserazide hcl	Dopamine	80
Plate 00016368	A8	Enalapril maleate	RAAS	78.94736
Plate 00016368	A9	Sitagliptin phosphate	DPP-4	78.94736
Plate 00016368	B1	Amlodipine besylate	calcium channel	50
Plate 00016368	B10	SB 743921	Kinesin spindle protein	77.19299
Plate 00016368	B11	ON-01910	PLK	80.35714
Plate 00016368	B12	Benazepril hydrochloride	RAAS	78.18182
Plate 00016368	B2	Imatinib Mesylate	c-Kit	72.88136
Plate 00016368	B3	BS-181 hydrochloride	CDK	75.86207

Plate 00016368	B4	KRN 633	VEGFR	78.94736
Plate 00016368	B5	Donepezil hydrochloride	mAChRs	80
Plate 00016368	B6	Perindopril Erbumine	RAAS	78.94736
Plate 00016368	B7	Chlorpromazine hcl	Dopamine	78.94736
Plate 00016368	B8	Clopidogrel bisulfate	NULL	78.94736
Plate 00016368	B9	Roscovitine(CYC202)	CDK	77.5862
Plate 00016368	C1	Bisoprolol Fumarate	Adrenergic receptors	77.19299
Plate 00016368	C10	Tropisetron hcl	5-HT receptor	76.27119
Plate 00016368	C11	R406	Syk	80.70175
Plate 00016368	C12	SNS-314 Mesylate	Aurora Kinase	77.9661
Plate 00016368	C2	Alendronate Sodium	farnesyl diphosphate synthase	78.57143
Plate 00016368	C3	Gemcitabine Hydrochloride	Antimetabolites	80.70175
Plate 00016368	C4	AZD8330	MEK	81.81818
Plate 00016368	C5	Diphenhydramine hcl	histamine H1	80.70175
Plate 00016368	C6	Asenapine maleate	5-HT	79.31035
Plate 00016368	C7	Doxazosin mesylate	adrenergic receptor	73.21429
Plate 00016368	C8	Pazopanib Hydrochloride	VEGFR	77.5862
Plate 00016368	C9	AS703026	MEK	78.94736
Plate 00016368	D1	PD153035 hydrochloride	EGFR	71.18644
Plate 00016368	D10	Ranitidine Hydrochloride	histamine H2-receptor	80.70175
Plate 00016368	D11	U0126-EtOH	MEK	80.35714
Plate 00016368	D12	AG-014699	PARP	53.57143
Plate 00016368	D2	Flavopiridol(Alvocidib)	CDK	21.68675
Plate 00016368	D3	Terbinafine hydrochloride	COX	81.03448
Plate 00016368	D4	Vicriviroc Malate	CCR5(cellular coreceptor 5)	78.94736
Plate 00016368	D5	Yohimbine hydrochloride	alpha 2-adrenergic receptors	80.35714
Plate 00016368	D6	Vincristine Sulfate	Microtubule Formation	75.86207
Plate 00016368	D7	KW 2449	Flt	75
Plate 00016368	D8	Adriamycin	Topoisomerase	91.07143
Plate 00016368	D9	BMS 794833	c-Met	75.4386
Plate 00016368	E1	PIK-75 Hydrochloride	PI3K	20.48193
Plate 00016368	E10	Dorzolamide HCL	carbonic anhydrase(CA)	80.35714
Plate 00016368	E11	Pramipexole dihydrochloride	NULL	81.81818
Plate 00016368	E12	PHA-793887	CDK	32.43243
Plate 00016368	E2	NVP-ADW742	IGF-1R	77.9661
Plate 00016368	E3	Amfebutamone hcl	norepinephrine-dopamine reuptake	82.14286
Plate 00016368	E4	Pancuronium bromide	mAChRs	78.57143
Plate 00016368	E5	Obatoclax Mesylate	Bcl-2	52.17391
Plate 00016368	E6	Vatalanib	VEGFR	78.57143
Plate 00016368	E7	BIX 02188	MEK	79.31035
Plate 00016368	E8	Selegiline hydrochloride	monoamine oxidase	80.35714
Plate 00016368	E9	Lapatinib Ditosylate	EGFR	71.18644
Plate 00016368	F1	17-DMAG	HSP90	75.86207
Plate 00016368	F10	Motesanib Diphosphate	VEGFR	78.57143
Plate 00016368	F11	MK-2206	Akt	71.92982
Plate 00016368	F12	PD0332991	CDK	75.86207
Plate 00016368	F2	Sunitinib Malate	FLT3	72.13115
Plate 00016368	F3	Fluoxetine hydrochloride	5-HT	80.70175
Plate 00016368	F4	Atropine sulfate monohydrate	mAChRs	82.45614
Plate 00016368	F5	Ketorolac Tromethamine	COX	82.14286
Plate 00016368	F6	Topotecan Hydrochloride	Topoisomerase	79.62963
Plate 00016368	F7	Pelitinib	EGFR	70.49181
Plate 00016368	F8	Venlafaxine hcl	5-HT receptor	80.35714
Plate 00016368	F9	Tamoxifen Citrate	Estrogen receptor	83.92857
Plate 00016368	G1	Nebivolol HCl	adrenergic receptor	75

Plate 00016368	G10	SGX-523	c-Met	80.35714
Plate 00016368	G11	MK 3207 hydrochloride	CGRP	80
Plate 00016368	G12	WZ8040	EGFR	80.35714
Plate 00016368	G2	YM155	Survivin	77.5862
Plate 00016368	G3	LY2228820	p38 MAPK	56.25
Plate 00016368	G4	BMS-599626	EGFR	74.13793
Plate 00016368	G5	HMN-214	PLK	80.70175
Plate 00016368	G6	Tamsulosin hydrochloride	hydroxysteroid dehydrogenase	82.14286
Plate 00016368	G7	Sorafenib Tosylate	VEGFR	71.92982
Plate 00016368	G8	Naftopidil Dihydrochloride	adrenergic receptor	81.81818
Plate 00016368	G9	ITF2357	HDAC	59.375
Plate 00016368	H1	DMSO	vehicle control	80
Plate 00016368	H2	DMSO	vehicle control	82.14286
Plate 00016368	H3	Cytochalasin D	actin	5.208333
Plate 00016368	H4	Cytochalasin D	actin	5.208333
Plate 00016503	A1	ENMD-2076	Flt	65.51724
Plate 00016503	A10	Alendronate Sodium	farnesyl diphosphate synthase	68.96552
Plate 00016503	A11	Flavopiridol(Alvocidib)	CDK	22.5
Plate 00016503	A12	NVP-ADW742	IGF-1R	70.17544
Plate 00016503	A2	Amlodipine besylate	calcium channel	68.42105
Plate 00016503	A3	Bisoprolol Fumarate	Adrenergic receptors	67.24138
Plate 00016503	A4	PD153035 hydrochloride	EGFR	61.66667
Plate 00016503	A5	PIK-75 Hydrochloride	PI3K	23.75
Plate 00016503	A6	17-DMAG	HSP90	60.65574
Plate 00016503	A7	Nebivolol HCl	adrenergic receptor	67.24138
Plate 00016503	A8	Sotalol hydrochloride	Beta-1 adrenergic receptor	70.68965
Plate 00016503	A9	Imatinib Mesylate	c-Kit	65.57377
Plate 00016503	B1	Sunitinib Malate	FLT3	63.93443
Plate 00016503	B10	Diclofenac sodium	COX	66.66666
Plate 00016503	B11	KRN 633	VEGFR	66.66666
Plate 00016503	B12	AZD8330	MEK	68.96552
Plate 00016503	B2	YM155	Survivin	65.57377
Plate 00016503	B3	Clemastine fumarate	histamine H1 antagonist	68.96552
Plate 00016503	B4	BS-181 hydrochloride	CDK	69.49152
Plate 00016503	B5	Gemcitabine Hydrochloride	Antimetabolites	70.68965
Plate 00016503	B6	Terbinafine hydrochloride	COX	67.24138
Plate 00016503	B7	Amfebutamone hcl	norepinephrine-dopamine reuptake	71.92982
Plate 00016503	B8	Fluoxetine hydrochloride	5-HT	69.49152
Plate 00016503	B9	LY2228820	p38 MAPK	48.52941
Plate 00016503	C1	Vicriviroc Malate	CCR5(cellular coreceptor 5)	70.68965
Plate 00016503	C10	Ketorolac Tromethamine	COX	70.17544
Plate 00016503	C11	HMN-214	PLK	67.79661
Plate 00016503	C12	WZ3146	EGFR	65
Plate 00016503	C2	Pancuronium bromide	mAChRs	71.66666
Plate 00016503	C3	Atropine sulfate monohydrate	mAChRs	70.68965
Plate 00016503	C4	BMS-599626	EGFR	60.65574
Plate 00016503	C5	Fasudil HCl	ROCK	67.79661
Plate 00016503	C6	Donepezil hydrochloride	mAChRs	67.24138
Plate 00016503	C7	Diphenhydramine hcl	histamine H1	69.49152
Plate 00016503	C8	Yohimbine hydrochloride	alpha 2-adrenergic receptors	69.49152
Plate 00016503	C9	Obatoclax Mesylate	Bcl-2	44.28571
Plate 00016503	D1	Perindopril Erbumine	RAAS	71.18644
Plate 00016503	D10	KW 2449	Flt	67.79661
Plate 00016503	D11	BIX 02188	MEK	69.49152
Plate 00016503	D12	Pelitinib	EGFR	55.55556

Plate 00016503	D2	Asenapine maleate	5-HT	68.42105
Plate 00016503	D3	Vincristine Sulfate	Microtubule Formation	66.10169
Plate 00016503	D4	Vatalanib	VEGFR	67.79661
Plate 00016503	D5	Topotecan Hydrochloride	Topoisomerase	71.92982
Plate 00016503	D6	Tamsulosin hydrochloride	hydroxysteroid dehydrogenase	71.18644
Plate 00016503	D7	Benserazide hcl	Dopamine	70.17544
Plate 00016503	D8	Chlorpromazine hcl	Dopamine	68.96552
Plate 00016503	D9	Doxazosin mesylate	adrenergic receptor	62.71186
Plate 00016503	E1	Sorafenib Tosylate	VEGFR	62.71186
Plate 00016503	E10	Roscovitine(CYC202)	CDK	66.66666
Plate 00016503	E11	AS703026	MEK	70.68965
Plate 00016503	E12	BMS 794833	c-Met	64.40678
Plate 00016503	E2	Enalapril maleate	RAAS	70.68965
Plate 00016503	E3	Clopidogrel bisulfate	NULL	68.96552
Plate 00016503	E4	Pazopanib Hydrochloride	VEGFR	64.40678
Plate 00016503	E5	Adriamycin	Topoisomerase	61.66667
Plate 00016503	E6	Selegiline hydrochloride	monoamine oxidase	72.4138
Plate 00016503	E7	Venlafaxine hcl	5-HT receptor	72.4138
Plate 00016503	E8	Naftopidil Dihydrochloride	adrenergic receptor	71.92982
Plate 00016503	E9	Sitagliptin phosphate	DPP-4	71.18644
Plate 00016503	F1	Lapatinib Ditosylate	EGFR	62.90322
Plate 00016503	F10	SGX-523	c-Met	70.68965
Plate 00016503	F11	OSI-930	c-Kit	66.66666
Plate 00016503	F12	ON-01910	PLK	70.68965
Plate 00016503	F2	Tamoxifen Citrate	Estrogen receptor	73.21429
Plate 00016503	F3	ITF2357	HDAC	44.92754
Plate 00016503	F4	Erlotinib Hydrochloride	EGFR	67.24138
Plate 00016503	F5	SB 743921	Kinesin spindle protein	66.10169
Plate 00016503	F6	Tropisetron hcl	5-HT receptor	71.92982
Plate 00016503	F7	Ranitidine Hydrochloride	histamine H2-receptor	70.68965
Plate 00016503	F8	Dorzolamide HCL	carbonic anhydrase(CA)	72.4138
Plate 00016503	F9	Motesanib Diphosphate	VEGFR	70.68965
Plate 00016503	G1	R406	Syk	68.42105
Plate 00016503	G10	PHA-793887	CDK	34.24657
Plate 00016503	G11	PD0332991	CDK	67.24138
Plate 00016503	G12	WZ8040	EGFR	71.92982
Plate 00016503	G2	U0126-EtOH	MEK	70.68965
Plate 00016503	G3	Pramipexole dihydrochloride	NULL	71.92982
Plate 00016503	G4	MK-2206	Akt	73.68421
Plate 00016503	G5	MK 3207 hydrochloride	CGRP	72.4138
Plate 00016503	G6	Granisetron Hydrochloride	5-HT receptor	68.96552
Plate 00016503	G7	Benazepril hydrochloride	RAAS	71.92982
Plate 00016503	G8	SNS-314 Mesylate	Aurora Kinase	65.57377
Plate 00016503	G9	AG-014699	PARP	65.51724
Plate 00016503	H1	Cytochalasin D	actin	3.296703
Plate 00016503	H2	Cytochalasin D	actin	4.301075
Plate 00016503	H3	DMSO	vehicle control	71.92982
Plate 00016503	H4	DMSO	vehicle control	70.68966
Plate 00016504	A1	BAY 73-4506(Regorafenib)	c-Kit	71.42857
Plate 00016504	A10	Zibotentan(ZD4054)	ETA-receptor	78.57143
Plate 00016504	A11	WYE-354	mTOR	80
Plate 00016504	A12	XL765	PI3K	80
Plate 00016504	A2	MGCD-265	c-Met	74.54546
Plate 00016504	A3	Bosutinib(SKI-606)	Src	73.21429
Plate 00016504	A4	PHA-739358(Danusertib)	Aurora Kinase	80

Plate 00016504	A5	BIBF1120(Vargatef)	VEGFR	74.13793
Plate 00016504	A6	AT9283	Bcr-Abl	71.42857
Plate 00016504	A7	CI-1033(Canertinib)	EGFR	68.96552
Plate 00016504	A8	Dasatinib	SRC	70.17544
Plate 00016504	A9	Camptothecine	Topoisomerase	76.36364
Plate 00016504	B1	WZ4002	EGFR	75
Plate 00016504	B10	Raltitrexed(Tomudex)	Antimetabolites	71.42857
Plate 00016504	B11	Epothilone B(EPO906)	Epothilone B	76.36364
Plate 00016504	B12	Vinorelbine(Navelbine)	p38 MAPK	72.72727
Plate 00016504	B2	MLN8237	Aurora	76.78571
Plate 00016504	B3	Cediranib(AZD2171)	VEGFR	70.68965
Plate 00016504	B4	ZM-447439	Aurora Kinase	71.42857
Plate 00016504	B5	Nilotinib	Bcr-Abl	73.68421
Plate 00016504	B6	AT7519	CDK	23.07692
Plate 00016504	B7	Masitinib(AB1010)	c-Kit	71.42857
Plate 00016504	B8	Gefitinib(Iressa)	EGFR	68.96552
Plate 00016504	B9	AZD1480	JAK	74.54546
Plate 00016504	C1	CCT129202	Aurora kinase	78.94736
Plate 00016504	C10	CHIR-99021	GSK-3	74.13793
Plate 00016504	C11	BMS 777607	c-Met	80
Plate 00016504	C12	Docetaxel	Microtubule Formation	77.19299
Plate 00016504	C2	GSK461364	PLK	79.62963
Plate 00016504	C3	PF-2341066	c-Met	76.36364
Plate 00016504	C4	BI 2536	PLK	71.18644
Plate 00016504	C5	TSU-68	VEGFR	80
Plate 00016504	C6	MP-470	c-Met	53.96825
Plate 00016504	C7	Tandutinib (MLN518)	FLT-3	78.57143
Plate 00016504	C8	Indirubin	CDK	81.81818
Plate 00016504	C9	Capecitabine(Xeloda)	Antimetabolites	79.62963
Plate 00016504	D1	XL184	VEGFR	80.35714
Plate 00016504	D10	AZD8055	mTOR	76.92308
Plate 00016504	D11	RAF265	RAF	78.18182
Plate 00016504	D12	Everolimus(RAD001)	mTOR	79.62963
Plate 00016504	D2	Neratinib	HER2	68.96552
Plate 00016504	D3	SB 525334	ALK	79.62963
Plate 00016504	D4	AP24534	VEGFR	54.6875
Plate 00016504	D5	CYC116	Aurora Kinase	77.19299
Plate 00016504	D6	AV-951(Tivozanib)	VEGFR	73.21429
Plate 00016504	D7	Vandetanib	VEGFR	77.19299
Plate 00016504	D8	BIBR1532	Telomerase	75.4386
Plate 00016504	D9	Fludarabine Phosphate	Antimetabolites	79.62963
Plate 00016504	E1	PHA-680632	Aurora Kinase	83.63636
Plate 00016504	E10	LY2784544	JAK	79.62963
Plate 00016504	E11	BIRB 796	p38 MAPK	68.96552
Plate 00016504	E12	Deforolimus(MK-8669)	mTOR	80.76923
Plate 00016504	E2	SU11274(PKI-SU11274)	c-Met	70.17544
Plate 00016504	E3	AZD7762	CHK	75
Plate 00016504	E4	XL880(GSK1363089)	c-Met	76.36364
Plate 00016504	E5	PF-04217903	c-Met	78.18182
Plate 00016504	E6	R406(free base)	Syk	81.81818
Plate 00016504	E7	VX-680	Aurora Kinase	79.62963
Plate 00016504	E8	GDC-0879	B-Raf	82.14286
Plate 00016504	E9	ABT-751	Microtubule Formation	81.48148
Plate 00016504	F1	AZD1152-HQPA(Barasertib)	Aurora Kinase	81.48148
Plate 00016504	F10	Pemetrexed disodium	TS	79.62963

Plate 00016504	F11	KU-60019	ATM	76.36364
Plate 00016504	F12	Enzastaurin	PKC	80
Plate 00016504	F2	Aurora A Inhibitor I	Aurora Kinase	71.92982
Plate 00016504	F3	Ki8751	VEGFR	81.13207
Plate 00016504	F4	Axitinib	VEGFR	83.33334
Plate 00016504	F5	JNJ-38877605	c-Met	81.81818
Plate 00016504	F6	AC-220	FLT	79.24529
Plate 00016504	F7	Quercetin(Sophoretin)	PI3K	79.24529
Plate 00016504	F8	NPI-2358	VDA	75.86207
Plate 00016504	F9	PIK-293	PI3K	82.14286
Plate 00016504	G1	Hesperadin	Aurora kinase	67.79661
Plate 00016504	G10	Epothilone A	Microtubule Formation	79.62963
Plate 00016504	G11	PD318088	MEK	81.48148
Plate 00016504	G12	Rapamycin(Sirolimus)	mTOR	79.62963
Plate 00016504	G2	NVP-TAE684	ALK	77.77778
Plate 00016504	G3	CP-724714	HER2	77.35849
Plate 00016504	G4	AZD0530(Saracatinib)	SRC	78.57143
Plate 00016504	G5	SNS-032(BMS-387032)	CDK	26.92308
Plate 00016504	G6	AZD6244(Selumetinib)	MEK	81.81818
Plate 00016504	G7	Imatinib(STI571)	PDGFR	78.57143
Plate 00016504	G8	AT7867	Akt	75
Plate 00016504	G9	AZD6482	PI3K	81.81818
Plate 00016504	H1	Cytochalasin D	actin	4.878049
Plate 00016504	H2	Cytochalasin D	actin	4.301075
Plate 00016504	H3	DMSO	vehicle control	80
Plate 00016504	H4	DMSO	vehicle control	81.48148
Plate 00016505	A1	Temsirolimus	mTOR	72.4138
Plate 00016505	A10	Maraviroc(Selzentry)	CCR5(cellular coreceptor 5)	74.13793
Plate 00016505	A11	Danoprevir(ITMN-191)	Proteasome	74.57627
Plate 00016505	A12	Lenalidomide	TNF-alpha	75.86207
Plate 00016505	A2	TGX-221	PI3K	67.79661
Plate 00016505	A3	Etoposide(Etopophos)	Topoisomerase	71.18644
Plate 00016505	A4	VX-702	p38 MAPK	60.31746
Plate 00016505	A5	LY294002	PI3K	69.49152
Plate 00016505	A6	Cytarabine(Cytosar-U)	DNA polymerase	72.4138
Plate 00016505	A7	Belinostat(PXD101)	HDAC	55.55556
Plate 00016505	A8	S31-201	STAT3	72.4138
Plate 00016505	A9	CUDC-101	HDAC	62.29508
Plate 00016505	B1	Decitabine	Antimetabolites	74.13793
Plate 00016505	B10	Ispinesib mesilate	Kinesin spindle protein	74.13793
Plate 00016505	B11	Ritonavir	HIV protease	75.4386
Plate 00016505	B12	MS-275	HDAC	77.19299
Plate 00016505	B2	IC-87114	PI3K	73.68421
Plate 00016505	B3	SB 431542	ALK	67.79661
Plate 00016505	B4	PLX-4720	B-Raf	71.92982
Plate 00016505	B5	SB 216763	GSK-3	74.13793
Plate 00016505	B6	EX 527	Sirtuin	75.4386
Plate 00016505	B7	BIIB021	HSP90	71.92982
Plate 00016505	B8	LAQ824	HDAC	50
Plate 00016505	B9	Raltegravir	HIV Integrase	75.4386
Plate 00016505	C1	AS-605240	PI3K	73.68421
Plate 00016505	C10	MLN9708	Proteasome	61.29032
Plate 00016505	C11	BMS-790052	HCV protease	70.68965
Plate 00016505	C12	NVP-AUY922	HSP90	71.92982
Plate 00016505	C2	ZSTK474	PI3K	72.4138

Plate 00016505	C3	PD98059	MEK	72.4138
Plate 00016505	C4	Irinotecan	topoisomerase	65.51724
Plate 00016505	C5	BIX 02189	MEK	73.68421
Plate 00016505	C6	Pomalidomide	TNF-alpha	75
Plate 00016505	C7	AG14361	PARP	73.21429
Plate 00016505	C8	JNJ-26481585	HDAC	53.84615
Plate 00016505	C9	VX-222	HCV protease	77.19299
Plate 00016505	D1	Cladribine	Antimetabolites	75.4386
Plate 00016505	D10	BMS-708163	¦Ã-secretase	76.36364
Plate 00016505	D11	ABT-888	PARP	75
Plate 00016505	D12	Trichostatin A	HDAC	68.33334
Plate 00016505	D2	GSK429286A	ROCK	72.88136
Plate 00016505	D3	Fludarabine(Fludara)	Antimetabolites	76.36364
Plate 00016505	D4	Paclitaxel(Taxol)	Microtubule Formation	71.66666
Plate 00016505	D5	GDC-0941	PI3K	74.13793
Plate 00016505	D6	LY500307	Er¦Â	75.86207
Plate 00016505	D7	JNJ 26854165	p53	71.42857
Plate 00016505	D8	MGCD0103(Mocetinostat)	HDAC	76.36364
Plate 00016505	D9	Elvitegravir	HIV Integrase	75
Plate 00016505	E1	Clofarabine	ribonucleotide reductase	75.86207
Plate 00016505	E10	Dutasteride(Avodart)	5¦Á-reductase	78.57143
Plate 00016505	E11	AZD2281(Olaparib)	PARP	75.4386
Plate 00016505	E12	Flucytosine(Ancobon)	DNA/RNA synthesis	75.4386
Plate 00016505	E2	VX-745	p38 MAPK	67.79661
Plate 00016505	E3	2-Methoxyestradiol	HIF	73.68421
Plate 00016505	E4	CI-1040 (PD184352)	MEK	75
Plate 00016505	E5	SB 202190	p38 MAPK	68.33334
Plate 00016505	E6	BSI-201	PARP	78.57143
Plate 00016505	E7	SB939	HDAC	61.29032
Plate 00016505	E8	BMS-707035	HIV-1 Integrase(IN)	78.18182
Plate 00016505	E9	RO4929097	Y-Secretase	76.36364
Plate 00016505	F1	TG100-115	PI3K	77.77778
Plate 00016505	F10	17-AAG(Geldanamycin)	HSP90	71.92982
Plate 00016505	F11	Bortezomib	proteasome	60.65574
Plate 00016505	F12	Droxinostat	HDAC	80
Plate 00016505	F2	XL147	PI3K	77.19299
Plate 00016505	F3	PIK-93	PI3K	74.54546
Plate 00016505	F4	PD0325901	MEK	78.18182
Plate 00016505	F5	SB 203580	p38 MAPK	74.13793
Plate 00016505	F6	Cryptotanshinone	Stat	78.94736
Plate 00016505	F7	MLN2238	Proteasome	62.29508
Plate 00016505	F8	Cyclopamine	Hedgehog	78.57143
Plate 00016505	F9	NVP-BEP800	HSP90	71.42857
Plate 00016505	G1	PIK-90	PI3K	77.19299
Plate 00016505	G10	Atazanavir(BMS-232632)	Proteasome	76.36364
Plate 00016505	G11	LBH-589(Panobinostat)	HDAC	53.125
Plate 00016505	G12	Ketoprofen(Actron)	COX	77.19299
Plate 00016505	G2	KU-0063794	mTOR	76.78571
Plate 00016505	G3	KU-55933	ATM	73.21429
Plate 00016505	G4	SP600125	JNK	73.68421
Plate 00016505	G5	Sodium valproate	HDAC	78.57143
Plate 00016505	G6	MC1568	HDAC	75.86207
Plate 00016505	G7	Semagacestat	Y-Secretase	78.57143
Plate 00016505	G8	GDC-0449(Vismodegib)	Hedgehog	78.18182
Plate 00016505	G9	Tipifarnib	farnesyltransferase(Ftase)	78.18182

Plate 00016505	H1	Cytochalasin D	actin	3.157895
Plate 00016505	H2	Cytochalasin D	actin	4.210526
Plate 00016505	H3	DMSO	vehicle control	79.62963
Plate 00016505	H4	DMSO	vehicle control	78.57143
Plate 00016506	A1	Mizoribine	DNA/RNA synthesis	70
Plate 00016506	A10	SGI-1776	Pim	68.96552
Plate 00016506	A11	Mitoxantrone	Topoisomerase	57.1
Plate 00016506	A2	Vorinostat	HDAC	55.38462
Plate 00016506	A3	Piroxicam	COX	67.79661
Plate 00016506	A4	Chlorprothixene	5-HT	70
Plate 00016506	A5	Fluvastatin Sodium	HMG-CoA reductase	66.10169
Plate 00016506	A6	Manidipine	calcium channel	66.10169
Plate 00016506	A7	Eplerenone	RAAS	68.96552
Plate 00016506	A8	Linezolid	NULL	69.49152
Plate 00016506	A9	Sulfanilamide	PABA	70.68965
Plate 00016506	B1	Letrozole	Aromatase	70
Plate 00016506	B10	AEE788	EGFR	57.37705
Plate 00016506	B11	ABT-869	RTK	62.29508
Plate 00016506	B2	Ibuprofen	COX	70.68965
Plate 00016506	B3	ABT-737	Bcl-2	68.42105
Plate 00016506	B4	Febuxostat	XAO	70
Plate 00016506	B5	Rivaroxaban(Xarelto)	Factor Xa	68.85246
Plate 00016506	B6	BIBR-1048	thrombin	66.66666
Plate 00016506	B7	Ramipril	RAAS	70.68965
Plate 00016506	B8	Felodipine	calcium channel	67.24138
Plate 00016506	B9	Carbamazepine(Carbatrol)	sodium channel	67.24138
Plate 00016506	C1	Anastrozole(Arimidex)	Aromatase	72.4138
Plate 00016506	C10	AG-490	JAK	71.66666
Plate 00016506	C11	BEZ235(NVP-BEZ235)	mTOR	73.21429
Plate 00016506	C2	XAV-939	Wnt	68.42105
Plate 00016506	C3	ABT-263(Navitoclax)	Bcl-2	70.68965
Plate 00016506	C4	Trilostane	hydroxysteroid dehydrogenase	72.4138
Plate 00016506	C5	Baicalin	prolyl endopeptidase	71.18644
Plate 00016506	C6	Icariin	PDE	68.96552
Plate 00016506	C7	Irbesartan(Avapro)	RAAS	71.92982
Plate 00016506	C8	Zalcitabine	NART	71.92982
Plate 00016506	C9	Pyrimethamine	dihydrofolate reductase(DHFR)	71.18644
Plate 00016506	D1	Exemestane	aromatase enzyme	71.18644
Plate 00016506	D10	BIBW2992(Tovok)	EGFR	61.40351
Plate 00016506	D11	PI-103	PI3K	70.68965
Plate 00016506	D2	Bicalutamide(Casodex)	androgen receptor	70.17544
Plate 00016506	D3	Allopurinol	xanthine oxidase	74.13793
Plate 00016506	D4	Pimobendan(Vetmedin)	PDE	70
Plate 00016506	D5	Apixaban	Factor Xa	70.90909
Plate 00016506	D6	S-(+)-Rolipram	PDE	72.4138
Plate 00016506	D7	Valsartan	RAAS	72.88136
Plate 00016506	D8	Cilostazol	PDE	72.88136
Plate 00016506	D9	Dipyridamole	PDE	68.96552
Plate 00016506	E1	Finasteride	5¦Á-reductase	73.68421
Plate 00016506	E10	Sulfamethoxazole	PABA	71.92982
Plate 00016506	E2	Fulvestrant	estrogen receptor	71.18644
Plate 00016506	E3	Stavudine	NART	71.92982
Plate 00016506	E4	Nisoldipine	calcium channel	71.42857
Plate 00016506	E5	BIBR 953	thrombin	71.92982
Plate 00016506	E6	Luteolin(Luteolol)	PDE	70.68965

Plate 00016506	E7	Telmisartan	RAAS	72.4138
Plate 00016506	E8	Amisulpride	Dopamine Receptor	73.68421
Plate 00016506	E9	Clozapine	5-HT1C receptor	66.10169
Plate 00016506	F1	CHR-2797	Aminopeptidase N(APN)	70.17544
Plate 00016506	F10	Apigenin	P450	67.24138
Plate 00016506	F2	INO-1001	PARP	72.4138
Plate 00016506	F3	Emtricitabine	nucleoside reverse transcriptase	71.42857
Plate 00016506	F4	Tadalafil(Cialis)	PDE	73.21429
Plate 00016506	F5	LDE225	Smo	71.42857
Plate 00016506	F6	Nizatidine	histamine H2-receptor	74.57627
Plate 00016506	F7	Carbidopa	aromatic-L-amino-acid decarboxylase	75.4386
Plate 00016506	F8	Naftopidil	¦Á1-adrenergic receptor	68.42105
Plate 00016506	F9	GSK1059615	PI3K	71.92982
Plate 00016506	G1	TW-37	Bcl-2	74.54546
Plate 00016506	G10	JNJ-7706621	CDK	65
Plate 00016506	G2	Acetaminophen(Tylenol)	COX	72.4138
Plate 00016506	G3	Tenofovir	NART	73.68421
Plate 00016506	G4	VX-770	CFTR	70.68965
Plate 00016506	G5	Vardenafil(Vivanza)	PDE	73.68421
Plate 00016506	G6	Enalaprilat	RAAS	75
Plate 00016506	G7	Ozagrel	thromboxane A2 synthetase	73.21429
Plate 00016506	G8	Cilnidipine	calcium channe	70.17544
Plate 00016506	G9	Cilomilast(SB-207499)	PDE	74.57627
Plate 00016506	H1	Cytochalasin D	actin	2.105263
Plate 00016506	H2	Cytochalasin D	actin	2.083333
Plate 00016506	H3	DMSO	vehicle control	75
Plate 00016506	H4	DMSO	vehicle control	74.54545
